# DrugRepPT: a deep pretraining and fine-tuning framework for drug repositioning based on drug’s expression perturbation and treatment effectiveness

**DOI:** 10.1093/bioinformatics/btae692

**Published:** 2024-11-19

**Authors:** Shuyue Fan, Kuo Yang, Kezhi Lu, Xin Dong, Xianan Li, Qiang Zhu, Shao Li, Jianyang Zeng, Xuezhong Zhou

**Affiliations:** Department of Artificial Intelligence, Beijing Key Lab of Traffic Data Analysis and Mining, School of Computer Science & Technology, Beijing Jiaotong University, Beijing 100044, China; Department of Artificial Intelligence, Beijing Key Lab of Traffic Data Analysis and Mining, School of Computer Science & Technology, Beijing Jiaotong University, Beijing 100044, China; Faculty of Engineering and IT, Australian AI Institute, University of Technology Sydney, Sydney, NSW 2007, Australia; Department of Artificial Intelligence, Beijing Key Lab of Traffic Data Analysis and Mining, School of Computer Science & Technology, Beijing Jiaotong University, Beijing 100044, China; Department of Artificial Intelligence, Beijing Key Lab of Traffic Data Analysis and Mining, School of Computer Science & Technology, Beijing Jiaotong University, Beijing 100044, China; Department of Artificial Intelligence, Beijing Key Lab of Traffic Data Analysis and Mining, School of Computer Science & Technology, Beijing Jiaotong University, Beijing 100044, China; Department of Automation, MOE Key Laboratory of Bioinformatics/Bioinformatics Division, BNRIST, Institute for TCM-X, Tsinghua University, Beijing 100084, China; School of Engineering, Westlake University, Hangzhou 310030, China; Department of Artificial Intelligence, Beijing Key Lab of Traffic Data Analysis and Mining, School of Computer Science & Technology, Beijing Jiaotong University, Beijing 100044, China

## Abstract

**Motivation:**

Drug repositioning (DR), identifying novel indications for approved drugs, is a cost-effective strategy in drug discovery. Despite numerous proposed DR models, integrating network-based features, differential gene expression, and chemical structures for high-performance DR remains challenging.

**Results:**

We propose a comprehensive deep pretraining and fine-tuning framework for DR, termed DrugRepPT. Initially, we design a graph pretraining module employing model-augmented contrastive learning on a vast drug–disease heterogeneous graph to capture nuanced interactions and expression perturbations after intervention. Subsequently, we introduce a fine-tuning module leveraging a graph residual-like convolution network to elucidate intricate interactions between diseases and drugs. Moreover, a Bayesian multiloss approach is introduced to balance the existence and effectiveness of drug treatment effectively. Extensive experiments showcase the efficacy of our framework, with DrugRepPT exhibiting remarkable performance improvements compared to SOTA (state of the arts) baseline methods (improvement 106.13% on Hit@1 and 54.45% on mean reciprocal rank). The reliability of predicted results is further validated through two case studies, i.e. gastritis and fatty liver, via literature validation, network medicine analysis, and docking screening.

**Availability and implementation:**

The code and results are available at https://github.com/2020MEAI/DrugRepPT.

## 1 Introduction

Drug development constitutes an indispensable part of disease treatment processes. However, it is a laborious, costly, and time-intensive endeavor (Chong and Sullivan 2007, Paul *et al.* 2010). Studies have shown that developing a new drug typically spans 17–20 years and incurs costs exceeding 2 billion dollars (Chan *et al.* 2019). As a promising strategy within drug development, drug repositioning (DR) aims to unveil new therapeutic applications for approved drugs. This approach holds the potential to expedite the pace of drug development significantly (Li *et al.* 2016, Jourdan *et al.* 2020), enhances the medical value of existing drugs, and provides new treatment options for complex diseases that lack effective therapies (Parvathaneni *et al.* 2019, Tanoli *et al.* 2021).

Typically, a DR strategy consists of three steps: identifying a candidate drug for a given disease; mechanistic assessment of the drug effect in preclinical models; and evaluation of efficacy in Phase II clinical trials (Pushpakom *et al.* 2019). The above steps show that the drug–disease association prediction problem involves four features and data: chemical structure of drugs, differential gene expression of drugs and diseases, drug–disease relationships, and effectiveness comparative relationships (ECR) among drugs which refers to the effectiveness comparison of different drugs for the treatment of the same disease (Yang *et al.* 2023). Therefore, the mainstream method of existing DR computational models is to construct heterogeneous networks of drugs and diseases, and then use network analysis algorithms to extract the features of drugs and diseases, which can capture different levels of information (Lotfi Shahreza *et al.* 2018). For example, Li *et al.* proposed a DR method NIMCGCN that used graph convolutional networks (GCNs) to capture their features from miRNA-disease similarity networks and finally inferred unknown drug–disease associations using neural inductive matrix completion. NIMCGCN uses structural information and nonlinear architecture to improve performance significantly (Li *et al.* 2020). Yu *et al.* (2021) proposed a DR method LAGCN, constructing a heterogeneous drug–disease network and applying GCN to predict drug–disease relationships. LAGCN combining the embeddings by the attention mechanism can improve the prediction performance. Network-based methods often encounter the limitation of insufficient biological data. Some DR methods take chemical structure or differential gene expression as prior knowledge and then use heterogeneous networks to capture potential information on drugs and diseases (Yang *et al.* 2022). For example, Liu *et al.* constructed a DR model termed HNet-DNN, which used a deep neural network, to predict drug–disease associations based on the features extracted from the drug–disease heterogeneous network with the ontology features of drugs and diseases. The low-dimensional features of HNet-DNN alleviate the overfitting problem (Liu *et al.* 2020). Cui *et al.* proposed a DR model GraphRepur that used drug-exposure gene expression and drug–drug link information as inputs to predict new drugs for breast cancer through a graph neural network (GNN). GraphRepur combines ontology and structural features with complementary advantages (Cui *et al.* 2021). Some DR methods that combine biological and structural features suffer from the problem of over-smoothing or weak generalization ability. There are only a few methods that use ECR among drugs to describe quantitatively the relationship between drugs and diseases. For example, Yang *et al.* proposed a DR method termed DRONet that considered ECR among drugs by combining network embedding (NE) and learning to rank. DRONet is the first model to consider ECR among drugs (Yang *et al.* 2023). Maria *et al.* proposed PlaNet for predicting drug efficacy by reasoning over a massive clinical knowledge graph. PlaNet is scalable, which allows obtaining predictions for new drugs (Brbic *et al.* 2024). Some methods are limited by the size of drug effectiveness datasets. Among the above models, for the mean reciprocal rank (MRR) and top 10 hit ratio (Hit@10), DRONet achieves the best performance (MRR = 0.5871 and Hit@10 = 0.2707). For the area under the precision-recall curve (AUPR), GraphRepur achieves the best performance (AUPR = 0.59), followed by LAGCN (AUPR = 0.3168) and NIMCGCN (AUPR = 0.2002).

The existing DR methods highlight the features needed to cover the entire process, i.e. the known drug–disease relationships and chemical structure of drugs before treatment, the mechanism of action of drugs during treatment, and the clinical efficacy evaluation after treatment, all of which are indispensable for predicting candidate drugs (Jarada *et al.* 2020). The primary challenges in constructing a high-performance DR model by effectively integrating the above features are as follows. First, drug–disease relationship data are often very sparse, which significantly affects the learning of the node features within the network. Therefore, how to fully utilize static features, such as the chemical structure of drugs, dynamic gene-level changes induced by drug treatments, and the network structure of drugs and diseases, while overcoming the limitations of existing DR data, remains a challenge. Another significant challenge lies in the integration of clinical treatment effectiveness information of drugs. While previous DR methods typically rely on binary relationships between drugs and diseases for model training, overlooking the nuanced variations in treatment effectiveness hampers prediction accuracy. Therefore, effectively integrating these two types of supervisory information into DR model designs remains a challenging task crucial for enhancing prediction accuracy.

**Figure 1.**
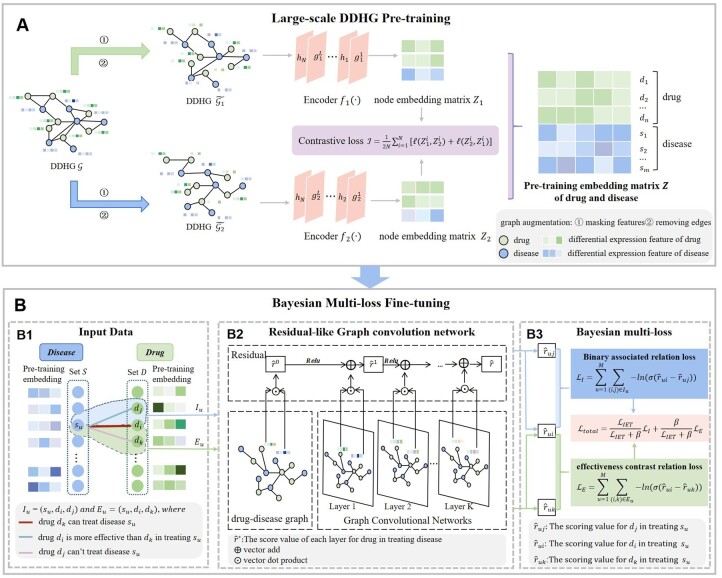
Overview of the proposed DrugRepPT. (A) A graph pretraining module with model-augmented contrastive learning on a large drug–disease heterogeneous graph. (B) A fine-tuning module of graph residual-like convolution network. (B1) The binary relationships between drugs and diseases and the ECR among drugs are used as supervisory information for model training. (B2) The graph residual-like convolution network to learn embedding features of drugs and diseases. (B3) Multiloss optimization with Bayesian averaging is used for drug prediction.

In this study, we propose DrugRepPT, a novel model for DR that incorporates a two-phase approach involving pretraining and fine-tuning to capture complex interactions among diseases and drugs (Fig. 1). In the pretraining phase, we construct a comprehensive drug–disease heterogeneous graph (DDHG) and initialize the node features by utilizing the information from differential gene expression following drug interventions. Through graph contrastive learning (GCL), we extract rich feature representations of drugs and diseases during pretraining. In the fine-tuning phase, the features obtained from pretraining are employed as inputs to a graph residual-like convolution network, designed to capture intricate interactions among drugs and diseases. Additionally, we introduce a Bayesian multiloss approach to balance effectively the existence and effectiveness of drug treatment. Comprehensive experimental evaluations and case analysis demonstrate the superior prediction accuracy and reliability of DrugRepPT compared to existing methods.

In summary, the main contributions of our work are four-fold.

- We propose a deep graph pretraining and fine-tuning framework for DR based on the drug’s features of gene expression perturbation and treatment effectiveness.
- We present a graph pretraining module with model-augmented contrastive learning on a large DDHG to capture nuanced interactions and expression perturbations postintervention.
- We introduce a fine-tuning module of graph residual-like convolution network to capture sophisticated interactions among diseases and drugs, along with a Bayesian multiloss to effectively balance binary relationships between drugs and diseases and ECR among drugs.
- The comprehensive experiments demonstrate that DrugRepPT performs better than the existing methods, and we take the two cases (i.e. gastritis and fatty liver) to demonstrate the reliability of predicted results, which are validated by biomedical literature and network medicine analysis, and used docking screening to find potential targets for the drug in treating disease.

## 2 Materials and methods

We propose DrugRepPT, a novel DR framework rooted in gene expression perturbation and ECR. Comprising two pivotal components, DrugRepPT offers a robust approach to DR. The first component entails a large-scale graph pretraining module, wherein we construct a comprehensive DDHG. Leveraging data on differential gene expression resulting from drug interventions, we utilize these expressions as node features for both drugs and diseases within the graph. We adeptly learn the intricate features of drugs and diseases through contrastive learning techniques applied to graphs. The second component, our fine-tuning module, utilizes the binary relationships between drugs and diseases and ECR among drugs as supervised information. To balance effectively these two sets of relationships, we introduce a Bayesian multiloss mechanism. Finally, employing a graph residual-like convolution network, we identify candidate drugs suitable for treating specific diseases, thereby showcasing the potential of DrugRepPT as an innovative framework in the field of DR.

### 2.1 Dataset

#### 2.1.1 Indication-oriented drug effectiveness comparative data

In our prior work, DRONet (Yang *et al.* 2023), we established a dataset of ECR among drugs through a combination of literature mining and hierarchical extension techniques. In this study, to obtain more accurate drug effectiveness data, we restricted the types of ECR among drugs and leveraged the drugs cataloged in the connectivity map (CMap) (Subramanian *et al.* 2017), which identified 234 additional drug relationships. Finally, through employing hierarchical extension techniques from DRONet, we expanded our dataset to encompass a total of 418 drug–disease relationships with effectiveness comparative information, enhancing the granularity and accuracy of our analysis. The details of the dataset and construction process are shown in Supplementary Section 1.1.1.

#### 2.1.2 Large-scale drug–disease heterogeneous graph

We have established a DDHG encompassing three kinds of relationships: drug–disease relationships from SemMed (Kilicoglu *et al.* 2012) and SIDER (Kuhn *et al.* 2016), drug–drug relationships from DrugBank (Wishart *et al.* 2018) and SIDER, and disease–disease relationships from SymMap (Wu *et al.* 2019) and SIDER. Finally, The constructed DDHG consists of 21 858 drugs, 2886 diseases, and 617 417 relationships (see details in Supplementary Section 1.1.2 and Supplementary Fig. S1A).

#### 2.1.3 Differential gene expression under drug intervention

Understanding gene expression levels is paramount because a drug in treating diseases is intricately linked to the expression or suppression of relevant genes. To establish the foundational features of drugs and diseases for DR modeling and subsequently enhance the precision of DR tasks, we leveraged gene expression perturbations observed under drug intervention from CMap. In this study, we focused on Level 5 data, which contains 1 319 138 signatures, corresponding to 25 200 biological entities and 473 647 experimental records. Subsequently, we meticulously screened and extracted 30 632 records about 19 811 compounds from this dataset. These records include essential details such as experiment ID, cell ID, and intervention specifics including compound ID, name, concentration, type, and duration, thus providing a comprehensive foundation for our research endeavors. The details of the data are shown in Supplementary Section 1.1.3.

### 2.2 Definition

In this section, we introduce related concepts of the heterogeneous and bipartite graphs and then formalize the DR problem.

Definition 1. DDHG. DDHG is a graph G=(V,E), where $E=(E_{\mathrm{drug}-\mathrm{drug}},E_{\mathrm{dis}-\mathrm{dis}},E_{\mathrm{drug}-\mathrm{dis}})$ denotes three kinds of relationships: drug–drug, disease–disease, and drug–disease, and V=(S,D) denotes the node set including all the drugs *S* and all the diseases *D*. Each node $v_{i}$ has an initial feature vector $f_{i}\in F\in R^{|V|\times N}$, where *N* is the dimensions of the feature.

Definition 2. Bipartite graph. We define the disease–drug bipartite graph as G=(V,E), where V=(S,D) represents the node set including all the drugs *S* and all the diseases *D*. Moreover, E⊂S×D denotes the edge set between *S* and *D*.

Definition 3. Problem definition. Given a DDHG G, the goal of pretraining is to train a model $f_{\mathrm{pre}}:G\to Z$ to predict embedding matrix *Z* of drugs and diseases. Then, given the disease–drug bipartite graph *G*, the goal of fine-tuning is to train a model *f*_fine_ to predict the score between each disease *s_u_* and each candidate drug *d_i_*, then rank the list of candidate drugs to obtain a predicted disease-related drug set.

### 2.3 Pretraining of large-scale DDHG

To fully account for changes in the protein molecular level of drug treatment of disease and address sparsity on known drug–disease relationships, we took the differential gene expression resulting from drug intervention as the features of drug and disease on DDHG, and we pretrained the model using unsupervised GCL methods to extract potential features of drugs and diseases.

#### 2.3.1 Gene expression features of drugs and diseases

We obtained differential gene expression generated under drug intervention from the CMap to improve the reliability of drug and disease profiles by emphasizing the mechanism of action when drugs treat disease. Specifically, for drugs recorded in CMap, their differential gene expression can be obtained directly; for drugs not recorded in CMap, we indirectly generated the differential gene expression of drugs by establishing the relationships between these drugs and the recorded drugs through the above drug similarity; for the drug-related diseases, we generated the differential gene expression of diseases based on the known disease–drug relationships. The details of the process are shown in Supplementary Section 1.2.1 and Supplementary Fig. S1B and C.

#### 2.3.2 Graph contrastive neural network for pretraining

To learn the complex relationship between diseases and drugs in DDHG, we designed a pretraining module with GCL, which can effectively obtain implicit knowledge of the embedding features of drugs and diseases. In the comparison study, we refer to model augmentation graph contrastive learning (MA-GCL) with excellent performance in the node classification task (Gong *et al.* 2023).

Specifically, for a given heterogeneous graph DDHG G, we use two graph data augmentation strategies (removing edges in the adjacency matrix and masking features for node attributes) to generate a pair of enhanced views $\left(G_{1},G_{2}\right)$, and then use the classical GNN as the two view encoders $\left(f_{1},f_{2}\right)$ to obtain node embedding $\left(Z_{1},Z_{2}\right)$ in the two views, with the final goal of minimizing the contrastive loss to distinguish the embeddings of the same node in these two different views from those of other nodes (Zhu *et al.* 2020). Subsequently, the model outputs embedding representation vectors for all drugs and diseases, which are then provided to the downstream DR fine-tuning model (see details in Supplementary Section 1.2.2).

### 2.4 Bayesian multiloss fine-tuning for DR

Because the existing DR methods only focus on the information of the binary relationships between drugs and diseases but do not consider the effectiveness information of drugs. Therefore, we designed a graph residual-like convolution network model as a fine-tuning model, integrated the above two relations as the supervision information of model training, and further introduced Bayesian multiloss to balance them, to improve the robustness and prediction accuracy of the model.

#### 2.4.1 Residual-like graph convolution network for fine-tuning

To achieve the downstream DR task, we designed a fine-tuning module of a graph residual-like convolution network. The model takes the bipartite graph formed by the known disease–drug relationships in the training set as the input graph, along with the embedding features of drug and disease derived from the pretraining model as the input features, and then uses the graph residual-like convolution network further to learn more accurate embedding features of drug and disease, and finally realizes drug prediction.

The graph residual-like convolution network first aggregates the node features to its neighbor nodes and uses a nonlinear transformation to update the node features. Finally, a residual-like structure is applied to mitigate the over-smoothing effect with deeper layers (see details in Supplementary Section 1.3.1).

#### 2.4.2 Multiloss optimization with Bayesian averaging

To fully optimize the fine-tuning model of residual-like GCN, we first considered both binary relationships between drugs and diseases and ECR among drugs as the supervised labels of the proposed model. Then we designed two objective functions based on pairwise relationships derived from the above two kinds of information. Finally, we introduced Bayesian averaging to generate position-aware weights (Hinne *et al.* 2020, Lu *et al.* 2023), resulting in balancing the two types of supervised information and obtaining a reliable DR fine-tuning model.

**Design of objective loss based on binary relationships between drugs and diseases**. The binary relationships between drugs and diseases refer to whether one marketed drug can treat the disease. Mathematically, for each disease $s_{u}$, we defined a triple $\left(s_{u},d_{i},d_{j}\right)$, where the drug $d_{i}$ (positive sample) treat the disease $s_{u}$, but $d_{j}$ (negative sample) cannot. Then, we trained the model to predict the scoring values ${\hat{r}}_{ui}$ and ${\hat{r}}_{uj}$ of $d_{i}$ and $d_{j}$ in treating $s_{u}$, respectively. Finally, we defined the loss function $L_{I}$, which encouraged ${\hat{r}}_{ui}$ to be higher than ${\hat{r}}_{uj}$:

(1) $$L_{I}=\sum_{u=1}^{M}\sum_{(i,j)\in I_{u}}-ln(\sigma ({\hat{r}}_{ui}-{\hat{r}}_{uj}))$$

where *M* is the number of diseases in the relationships dataset and *σ* is the activation function sigmoid (Yin *et al.* 2003).

**Design of objective loss based on ECR.** The ECR among drugs refers to when both drugs can treat a disease, with one exhibiting greater effectiveness than the other. Mathematically, for each disease $s_{u}$, we defined a triple $\left(s_{u},d_{i},d_{k}\right)$, where the drug $d_{i}$ is more effective than the drug $d_{k}$ in treating disease $s_{u}$. Then, we trained the model to predict the scoring values ${\hat{r}}_{ui}$ and ${\hat{r}}_{uk}$ of $d_{i}$ and $d_{k}$ in treating $s_{u}$, respectively. Finally, we defined the loss function $L_{E}$, which encouraged ${\hat{r}}_{ui}$ to be higher than ${\hat{r}}_{uj}$:

(2) $$L_{E}=\sum_{u=1}^{N}\sum_{(i,k)\in E_{u}}-ln(\sigma ({\hat{r}}_{ui}-{\hat{r}}_{uk}))$$

where *N* is the number of diseases in the effectiveness comparative dataset.

**Bayesian averaging to balance two objective losses**. To balance the weights of two optimization objectives, we designed a Bayesian average multiloss optimization, which adjusts the influence of two losses and combines them by changing position-aware weights β. The formula is as follows:

(3) $$L_{\mathrm{total}}=\frac{L_{\mathrm{IET}}}{L_{\mathrm{IET}}+\beta }L_{I}+\frac{\beta }{L_{\mathrm{IET}}+\beta }L_{E}+\lambda ||\Theta |{|}^{2}$$

where $L_{\text{IET}}=L_{I}+L_{E},\;||\Theta |{|}^{2}$ represents the L2 regularization applied to the neural network parameters, *λ* is a regularization parameter. *β* is the position-aware weight, and changing *β* can affect the gravity of the two losses. Specifically, when *β* approaches 0, Bayesian averaging focuses primarily on $L_{I}$, emphasizing the binary relationships between drugs and diseases during model optimization. When *β* approaches ∞, Bayesian averaging focuses more on $L_{E}$, highlighting the ECR among drugs during model optimization.

### 2.5 Experimental settings

In the experiments, we divided 2656 drug–disease relationships into the training and test set in an 8:2 ratio. The training set was used to train the model, while the test set was employed to evaluate the model’s performance (Supplementary Section 2.1). We selected two categories of baseline methods to compare with our proposed method and compared the input features of the baseline methods (Supplementary Section 2.2). The one is the existing DR methods, including LAGCN (Yu *et al.* 2021), NIMCGCN (Li *et al.* 2020), HNet-DNN (Liu *et al.* 2020), and DRONet (Yang *et al.* 2023). The other is the prediction methods based on embedding feature similarity, including singular value decomposition (SVD), high order proximity preserved embedding (HOPE) (Ou *et al.* 2016), graph factorization (GF) (Ahmed *et al.* 2013), GraRep (Cao *et al.* 2015), DeepWalk (Perozzi *et al.* 2014), node2vec (Grover and Leskovec 2016), large-scale information network embedding (LINE) (Tang *et al.* 2015), and structural deep network embedding (SDNE) (Wang *et al.* 2016). In addition, MRR and Hit@K used in DRONet (Yang *et al.* 2023) were selected as evaluation metrics and evaluated the performance improvement of DrugRepPT by using the relative improvement rate (Supplementary Section 2.3). We calculated the mean and standard deviation for each evaluation metric after 10 times repeated experiments, reporting the results as mean±standard deviation. Finally, the details of parameter settings are shown in Supplementary Section 2.4.

## 3 Results

In the experiments, to demonstrate the effectiveness of our framework, we compared the proposed DrugRepPT with multiple baseline methods on DR performance. Second, we designed ablation experiments to analyze the contribution of key components, objective loss, and different heterogeneous networks. Then, we compared the representation features to demonstrate interpretability and evaluated the robustness of the model through hyperparameter sensibility analysis. Finally, we evaluated the reliability of the model predictions in three ways, including validation of biomedical literature, analysis of network medicine, and docking-based virtual screening.

### 3.1 Overview of the performance comparison of DR methods

**Figure 2.**
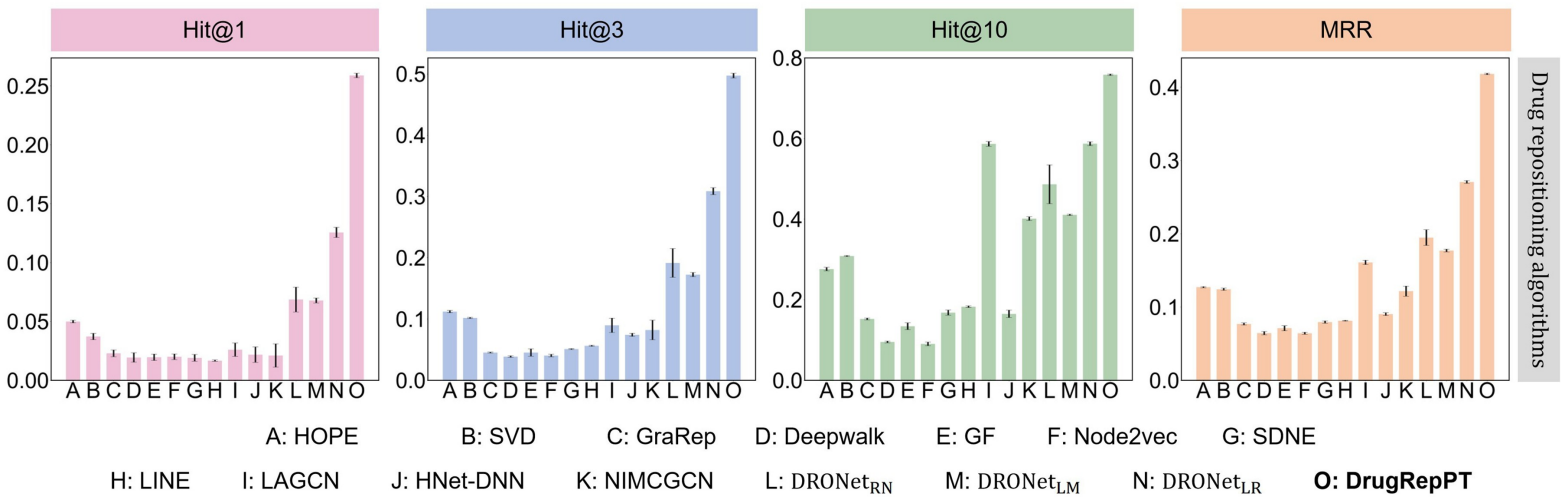
Performance comparison of drug repositioning algorithms. The error bars represent the standard deviation for each evaluation metric in 10 repeated experiments.

To conduct a comprehensive performance evaluation of DrugRepPT, we compared its predicted results with multiple baseline methods on DR performance (Table 1 and Fig. 2).

**Table 1.** Performance comparison of drug repositioning methods.

DR methods	Hit@1	Hit@3	Hit@10	MRR
HOPE	0.0499±0.0011	0.1121±0.0019	0.276±0.0043	0.1271±0.0008
SVD	0.0372±0.0027	0.1018±0.0009	0.3083±0.0012	0.1243±0.0015
GraRep	0.0229±0.0029	0.0453±0.0008	0.1523±0.0023	0.0769±0.0015
Deepwalk	0.0194±0.004	0.0387±0.0013	0.0952±0.0022	0.0644±0.0023
GF	0.0196±0.0027	0.0453±0.006	0.1341±0.0085	0.0711±0.0035
Node2vec	0.02±0.0024	0.0404±0.002	0.0903±0.0045	0.0642±0.0014
SDNE	0.0191±0.0029	0.051±0.0007	0.1679±0.0064	0.0795±0.0016
LINE	0.0167±0.0006	0.0563±0.0008	0.1826±0.0021	0.0813±0.0005
LAGCN	0.026±0.0057	0.0897±0.0117	0.5866±0.006	0.1609±0.0029
HNet-DNN	0.0218±0.0066	0.0742±0.0025	0.1649±0.0093	0.0904±0.0019
NIMCGCN	0.0211±0.0099	0.0821±0.016	0.4012±0.0047	0.1217±0.0069
*DRONet_RN_* ^a^	0.0686±0.0105	0.1915±0.0235	0.4863±0.0483	0.1948±0.0107
*DRONet_LM_* ^a^	0.0678±0.0021	0.1725±0.0034	0.4106±0.0016	0.1771±0.0019
*DRONet_LR_* ^a^	0.1256±0.0043	0.3086±0.0057	0.5871±0.0048	0.2707±0.0019
DrugRepPT	**0.2589±0.0019**	**0.4971±0.0039**	**0.7582±0.0018**	**0.4181±0.001**
Improvement^b^	106.13%	61.08%	29.14%	54.45%

± Represents a range of values, which preceded and followed by the mean and standard deviation after 10 times repeated experiments.

The best and second-best results are highlighted in boldface and underlined respectively.

a *DRONet_RN_*, *DRONet_LM_*, and *DRONet_LR_* denote three versions of DRONet based on different ranking learning algorithms, i.e. RankNet, LambdaMART, and LambdaRank.

b The relative improvement rate of DrugRepPT compared to the best baseline *DRONet_LR_*.

On the one hand, to evaluate the predictive performance of DrugRepPT, we compared the performance of DrugRepPT with all baseline methods. The results showed that DrugRepPT achieved the best performance (MRR = 0.4181 and Hit@10 = 0.7582). Specifically, the MRR and Hit@10 metrics of DrugRepPT are 54.45% and 29.14% higher than that of the best baseline (i.e. *DRONet_LR_*), indicating that the effective balance between the existence and effectiveness comparisons of drug treatment facilitates sophisticated interactions among diseases and drugs; for the best baseline based on GCN (i.e. LAGCN), DrugRepPT outperforms Hit@10 by 29.25%, suggesting that the residual-like mechanism can effectively solve the over-smoothing problem existing in GCN. In addition, DrugRepPT has achieved such excellent predictive performance because it accounts for gene expression perturbations under drug intervention, and combines pretraining based on GCL and fine-tuning model based on graph residual-like convolution network for DR prediction.

On the other hand, to analyze the specific reasons for the DrugRepPT performance advantage, we conducted an internal comparison of the two baseline models. The experimental results indicated that, among the baselines utilizing NE similarity, HOPE based on matrix factorization achieved the best performance (MRR = 0.1271 and Hit@1 = 0.0499), followed by SVD, and the SDNE based on neural network and based on neighborhood similarity performed the worst. In the existing DR models, *DRONet_LR_* attained the highest performance (MRR = 0.2707, Hit@10 = 0.5871), followed by *DRONet_RN_* (MRR = 0.1948, Hit@10 = 0.4863). The possible reason is that *DRONet_LR_* considers ECR among drugs, while other existing DR models rely solely on the binary relationships between drugs and diseases as supervisory information. In addition, in the MRR metric, the best baseline based on GCN (i.e. LAGCN) performs 26.59% better than the best baseline based on NE similarity, indicating that GCN facilitates superior aggregation of network topological information.

### 3.2 Ablation experiments of DrugRepPT

To evaluate the contribution of different parts in DrugRepPT, we conducted ablation experiments within DrugRepPT. Specifically, we analyze the contribution of key components, objective loss, and different heterogeneous networks. The details of the contribution of different heterogeneous networks are shown in Supplementary Section 3.1.1 and Supplementary Table S2.

#### 3.2.1 Contribution of key components of DrugRepPT

**Figure 3.**
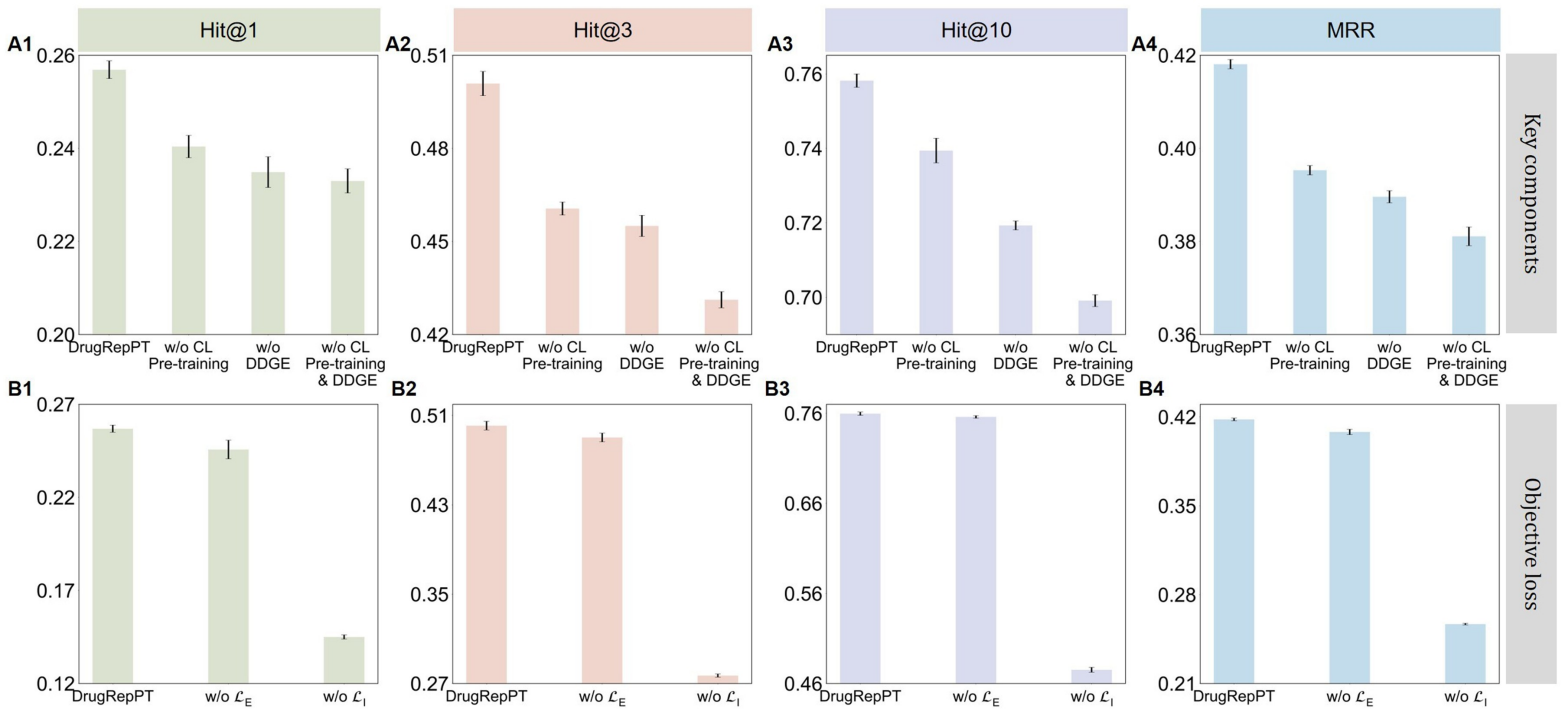
Performance comparison of ablation experiments of DrugRepPT. (A1–A4) Performance comparison of the contribution of key components. (B1–B4) Performance comparison of the contribution of objective loss.

To evaluate the contribution of different components in DrugRepPT, we conducted ablation experiments of DrugRepPT (Fig. 3A1–A4). Concretely, we compared our method DrugRepPT with three variants, defined as follows:

w/o CL pretraining: This variant removes pretraining based on GCL, using the features of gene expression of drug and disease (termed DDGE) as input to a downstream fine-tuning module to evaluate the contribution of pretraining.
w/o DDGE: This variant removes gene expression features from the pretraining module and uses randomly initialized features as input to the pretraining module to evaluate the contribution of DDGE features.
w/o CL pretraining and DDGE: This variant removes the pretraining module and corresponding gene expression features, using random initialization features conforming to the normal distribution as input to a downstream fine-tuning module to evaluate the contribution of the combination of DDGE and CL pretraining.

First, the results showed that the model performance decreases significantly when removing any of DrugRepPT’s components, indicating that both DDGE and CL pretraining positively contribute to the predictive performance. Second, compared with removing the pretraining module, the DrugRepPT performance decreased significantly after removing DDGE (decreased MRR = 6.57% for DDGE versus 5.20% for pretraining), indicating that DDGE has a greater contribution to improving the performance of the model. Finally, when both components were removed, DrugRepPT experienced the largest performance decline (8.61% MRR decline). This indicates that the combination of DDGE and CL pretraining has a synergistic improvement effect on the predictive performance of the model.

#### 3.2.2 Contribution of objective loss

To evaluate the contribution of different losses, we conducted ablation experiments on the fine-tuning part of DrugRepPT (Fig. 3B1–B4). Specifically, we compare the fine-tuning model of the DrugRepPT method with two variants, defined as follows:

w/o $L_{I}$: This variant removes the ECR among drugs loss from the fine-tuning module and retains only the drug treat disease loss to evaluate the contribution of the drug effectiveness comparative loss.
w/o $L_{E}$: This variant removes the drug treatment disease loss from the fine-tuning module and retains only the drug effectiveness comparative loss to evaluate the contribution of the drug treatment disease loss.

The results showed that model performance decreases significantly when any losses from the DrugRepPT fine-tuning model are removed, suggesting that both the drug effectiveness comparative loss and the drug treatment disease loss are effective in predicting performance. Second, DrugRepPT’s performance decreased more significantly after removing $L_{I}$ than after removing $L_{E}$ (decreased MRR: 1.58% for $L_{E}$ versus 62.38% for $L_{I}$).

### 3.3 Prediction of unknown diseases

To evaluate DrugRepPT’s ability to predict unknown diseases, we compared its predicted results against multiple baseline methods on DR performance. In the new-disease setting, diseases were divided into training diseases and test diseases at an 8:2 ratio. There are no overlapped diseases between the training diseases and test diseases. Once the DrugRepPT model was learned from the training data, which consists of the training diseases and the related drugs, it was used to predict relationships between test diseases and all the drugs. The baseline methods based on embedding feature similarity do not generate embedding for test diseases absent from the training set, making it difficult to predict drug candidates for unknown test diseases. Thus, we compared DrugRepPT with existing DR methods capable of predicting new diseases, including LAGCN, NIMCGCN, HNet-DNN, and DRONet, to evaluate performance (Table 2).

**Table 2.** Performance comparison of DR methods for unknown diseases.

DR methods	Hit@1	Hit@3	Hit@10	MRR
LAGCN	0.0083±0.0036	0.0294±0.0034	0.072±0.0034	0.0295±0.0022
HNet-DNN	0.0018±0.0026	0.0219±0.0025	0.0382±0.0093	0.0297±0.0019
NIMCGCN	0.0144±0.0025	0.0396±0.0033	0.0892±0.0029	0.0383±0.0017
*DRONet_RN_* ^a^	0.0512±0.0087	0.1410±0.0017	0.3063±0.0026	0.1362±0.0013
*DRONet_LM_* ^a^	0.0503±0.0019	0.1408±0.0032	0.2504±0.0011	0.1245±0.0017
*DRONet_LR_* ^a^	0.0515±0.0095	0.1417±0.0053	0.3137±0.0050	0.1385±0.0060
DrugRepPT	**0.0660±0.0019**	**0.1914±0.0030**	**0.4760±0.0015**	**0.1868±0.0015**
Improvement^b^	28.16%	35.07%	51.74%	34.87%

± Represents a range of values, which preceded and followed by the mean and standard deviation after 10 times repeated experiments.

The best and second-best results are highlighted in boldface and underlined respectively.

a *DRONet_RN_*, *DRONet_LM_*, and *DRONet_LR_* denote three versions of DRONet based on different ranking learning algorithms, i.e. RankNet, LambdaMART, and LambdaRank.

b The relative improvement rate of DrugRepPT compared to the best baseline *DRONet_LR_*.

The results showed that DrugRepPT achieved the best performance (MRR = 0.1868 and Hit@10 = 0.476), which is higher than all baseline methods. Specifically, the MRR and Hit@10 metrics are 34.87% and 51.74% higher than those of the best baseline (i.e. *DRONet_LR_*). This improvement likely be attributed to DrugRepPT’s diversified information, which is different from the sparse disease–drug relationships used in previous methods, DrugRepPT introduces the DDHG that includes the relationship of disease–drug, drug–drug, and disease–disease, and employs GCL to extract structural information, followed by a residual-like graph convolution network to obtain neighbor information. This combination of information helps alleviate the cold start problem to some extent. Consequently, DrugRepPT is suitable for predicting unknown diseases.

### 3.4 Interpretability of the representation features in DrugRepPT

**Figure 4.**
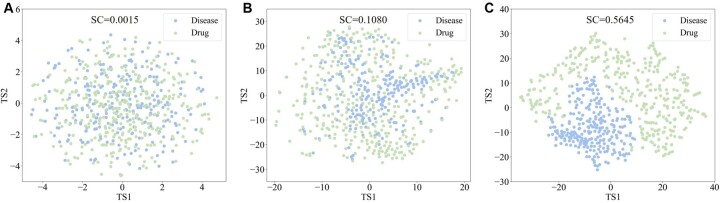
Visualization of the embedding features of drugs and diseases. (A) Visualization of embedding features from random initialization. (B) Visualization of embedding features from gene expression. (C) Visualization of embedding features from CL pretraining.

To visually compare the difference in features from gene expression, CL pretraining, and random initialization for diseases and drugs, we used t-SNE to visualize these features in two dimensions and calculate the silhouette score (SS) of node distribution (Fig. 4). The value interval of SS is [−1,1], and the larger the value, the stronger the aggregation of the node distribution.

The results showed that visualization results for both gene expression and CL pretraining features had higher clustering (SS = 0.1080 and SS = 0.5645) compared to random features (SS = 0.0014), where disease and drug were separated into two clusters. In addition, the visualization results of CL pretraining features showed greater aggregation than gene expression, with a more distinct separation between disease and drug clusters. This indicates that the pretraining method based on GCL can obtain more differentiated features of drug and disease, which is conducive to improving the accuracy of the DR task in the subsequent fine-tuning stage, as evidenced by the results of the ablation experiments above.

### 3.5 Hyperparameter sensibility analysis

To evaluate the robustness of our DrugRepPT, we conducted comprehensive experiments of hyperparameter sensibility analysis. The experiments involved fine-tuning hyperparameters such as position-aware weight *β*, learning rate, batch size, and the number of GCN layers. The details of the result and analysis are shown in Supplementary Fig. S2 and Supplementary Section 3.2.

### 3.6 Case analysis of gastritis and fatty liver

To illustrate the reliability of the predicted results of DrugRepPT, we selected two typical diseases, namely gastritis and fatty liver, and showed their top 10 drugs predicted by our model (Table 3). First, the results showed that omeprazole and rosiglitazone ranked first in the test sets for gastritis and fatty liver. Then the reliability of the left drugs is evaluated in three ways, including validation of biomedical literature, analysis of network medicine, and docking-based virtual screening.

**Table 3.** Top 10 candidate drugs predicted by DrugRepPT for gastritis and fatty liver.

	Gastritis		Fatty liver	
Rank	Predicted drug	Predicted validation	Predicted drug	Predicted validation
1	Omeprazole^a^	Test set	Rosiglitazone^a^	Test set
2	Cefixime^b^	Dahal *et al.*	Tacrolimus^b^	Carroll *et al.*
3	Ranitidine^b^	Madani *et al.*	Pyrazinamide	Docking (BE=−4.64)
4	Amikacin^b^	Quentin *et al.*	Ticlopidine^b^	Malladi *et al.*
5	Rofecoxib	Docking (BE=−6.82)	Montelukast^b^	Abdallah *et al.*
6	Prednisone^b^	Kharbanda *et al.*	Clarithromycin	Docking (BE=−15.64)
7	Tramadol	Docking (BE=−5.04)	Doxorubicin^b^	Porteiro *et al.*
7	Tramadol	Docking (BE=−5.04)	Doxorubicin^b^	Porteiro *et al.*
8	Clindamycin^b^	Matis *et al.*	Olanzapine	Docking (BE=−8.89)
9	Lenalidomide	Docking (BE=−6.0)	Urapidil	Docking (BE=−7.47)
10	Nimesulide	Docking (BE=−6.43)	Sulfasalazine	Docking (BE=−6.79)

a These candidate drugs predicted by DR models are in the test set.

b Several newly published literature reported that these candidate drugs are possibly related to the treatment of gastritis and fatty liver.

#### 3.6.1 Validation of biomedical literature

We retrieved the newly published biomedical literature from PubMed and evaluated the top 10 drugs predicted by DrugRepPT for gastritis and fatty liver.

The results showed that five drugs were mentioned in newly published literature concerning gastritis treatment. Specifically, Dahal *et al.* (2017) found that the most common infections treated with third-generation cephalosporins cefixime (rank = 2 in DrugRepPT) were acute gastritis. Madani *et al.* (2014) compared acid suppression (pharmacodynamics) and pharmacokinetics of IV famotidine and ranitidine (rank = 3 in DrugRepPT) in critically ill children at risk for stress gastritis. Quentin *et al.* found that an empirical antibiotic therapy with piperacillin/tazobactam and amikacin (rank = 4 in DrugRepPT) alleviated necrotizing esophagitis and gastritis caused by hypervirulent PVL positive ST 121 CA-MRSA through clinical cases (Richier *et al.* 2022). Kharbanda *et al.* (2023) analyzed medical records and found that patients with granulomatous gastritis significantly improved renal function and gastrointestinal symptoms with oral prednisone (rank = 6 in DrugRepPT). Matis *et al.* (2023) found that vancomycin and piperacillin-tazobactam administered with clindamycin (rank = 8 in DrugRepPT) improved the clinical presentation of emphysematous gastritis. The details of the analysis about fatty liver are shown in Supplementary Section 3.3.1.

In addition, the results of *DRONet_LR_* (the best baseline) showed that five drugs and three drugs were mentioned in newly published literature concerning the treatment of gastritis and fatty liver. The details of the result and analysis are shown in Supplementary Table S3 and Supplementary Section 3.3.1.

Literature validation results show that our DrugRepPT can obtain more reliable predictions than the baseline model, not only for some existing drugs but also for some new drug candidates.

#### 3.6.2 Validation of network medicine

**Figure 5.**
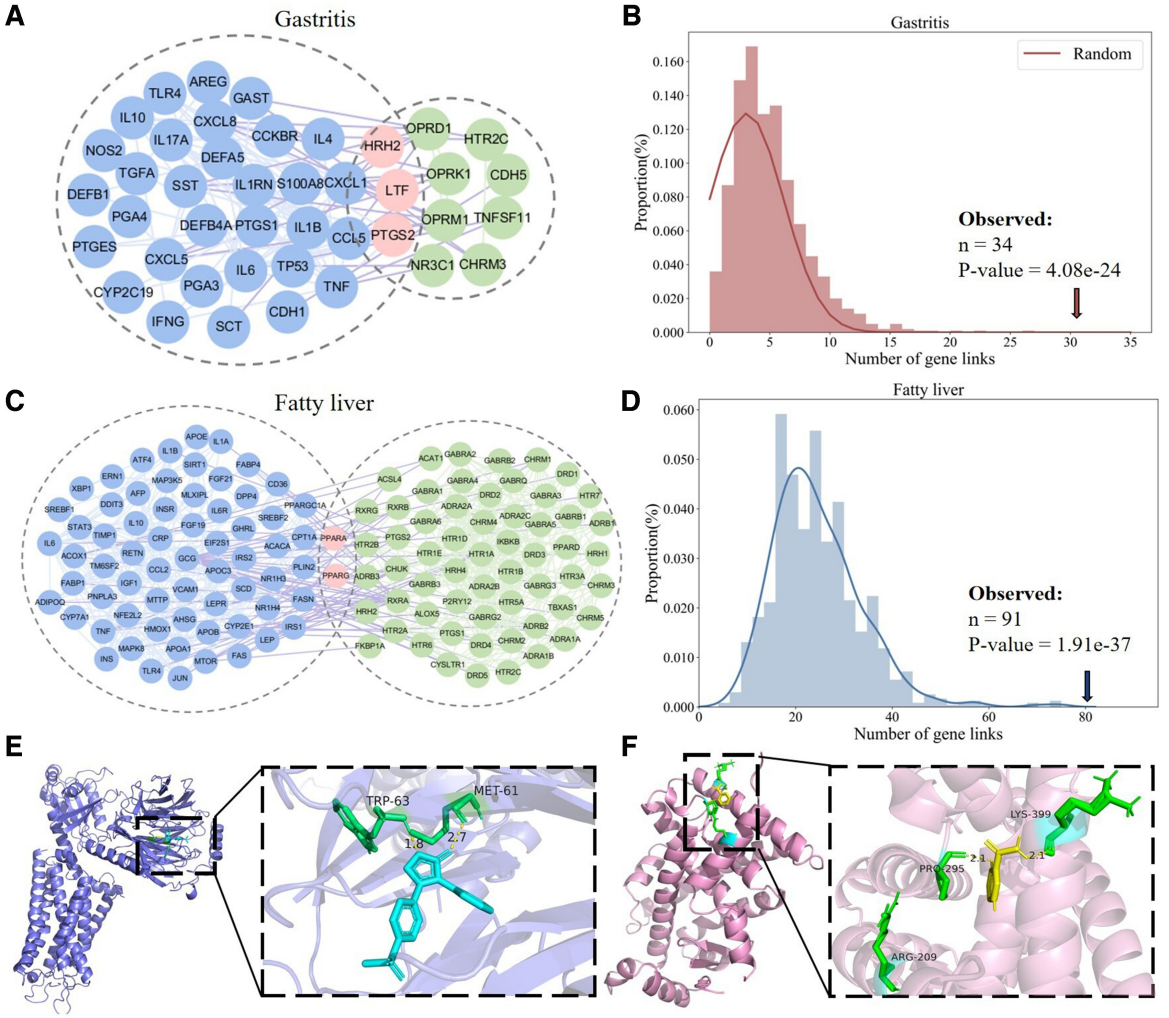
Network medicine-based molecular network association analysis and validation of docking-based virtual screening. (A) PPI network of the genes of gastritis (in the dashed circle on the left) and the targets of drugs (in the dashed circle on the right). The nodes in the intersection of the two dashed circles refer to common targets of drugs and gastritis. (B) Analysis of the high network closeness between the genes of gastritis and the targets of drugs. (C) PPI network of the genes of fatty liver (in the dashed circle on the left) and the targets of drugs (in the dashed circle on the right). The nodes in the intersection of the two dashed circles refer to common targets of drugs and fatty liver. (D) Analysis of the high network closeness between the genes of fatty liver and the targets of drugs. (E) Docking results between rofecoxib and the protein 7F8V of gastritis. (F) Docking results of pyrazinamide and the protein 6LXA of fatty liver.

The interaction between proteins often indicates the correlation of proteins in molecular functions or biological pathways. To validate the reliability of the candidate drugs predicted by our DrugRepPT, we conducted network medicine-based molecular network association analysis to measure the correlation between the genes of diseases and the targets of drugs. Specifically, we obtained the genes related to gastritis and fatty liver from the MalaCards (Rappaport *et al.* 2017) and the targets of these candidate drugs from the DrugBank (Wishart *et al.* 2018). After that, protein–protein interaction (PPI) network links between the disease genes and the corresponding drug targets were obtained and visualized from the STRING V11.5 database (Snel *et al.* 2000) (Fig 5A and C). Finally, to illustrate the high network closeness between the genes of diseases and the targets of drugs, we ran 1000 random simulations to generate PPI links between the disease genes and the corresponding drug targets and compared the number of observed connections with random controls. By calculating *P*-values using the binomial test, we obtained the probability of actual links to highlight the significance of the closeness between the genes of diseases and the targets of drugs.

The results illustrated the high network closeness between the genes of diseases and the targets of drugs for gastritis and fatty liver. The dense links (34 real links versus 3.07 expected links, *P* = 4.08E-24, binomial test) that hold in the PPI network between the 50 genes related to gastritis and the 28 targets of predicted drugs (Fig. 5B) indicated that those two kinds of genes would tend to interact more closely than expected. The details of the analysis about fatty liver are shown in Supplementary Section 3.3.2.

The analysis results of network medicine showcase that there is a high correlation and network closeness between the targets of the predicted drug and disease genes, which further reflects the reliability of DrugRepPT’s prediction.

#### 3.6.3 Validation of docking-based virtual screening

To verify the potential relationship between predicted drugs and diseases, we quantified the binding of drugs to disease-associated proteins by molecular docking. Specifically, we first retrieved the MalaCards database (Rappaport *et al.* 2017) to obtain the genes with the highest scores associated with gastritis and fatty liver, namely GAST and PPARA. Then we retrieved the PDB database and obtained the protein 7F8V contained in the gene GAST related to gastritis and the protein 6LXA contained in the gene PPARA related to fatty liver. We obtained the 3D structures of the two proteins from the PDB database and the 3D structures of these predicted drugs from the PubChem database (Wang *et al.* 2009). Finally, we performed molecular docking that considers predicted drugs as small molecules and the disease-related proteins as ligands using Autodock 1.5.7.

The results of molecular docking showed that there is strong binding energy (BE) with the protein 7F8V related to gastritis for the four predicted drugs, namely rofecoxib (ranked 5, BE=−6.82), tramadol (ranked 7, BE=−5.04), lenalidomide (ranked 9, BE=−6.0), and nimesulide (ranked 10, BE=−6.43) (Table 3). For example, rofecoxib was docked to the amino acid residue of 7F8V, namely TRP-63 and MET-61 (Fig. 5E). In addition, the details of the analysis about fatty liver are shown in Supplementary Section 3.3.3. The results of the docking analysis indicated a potential relationship between the predicted drugs and diseases.

In summary, we took gastritis and fatty liver as typical cases and validated the predicted drugs generated by DrugRepPT through comprehensive analysis, including biomedical literature, network medicine, and molecular docking. The results indicated that our DrugRepPT can obtain reliable prediction results, capable of not only identifying existing drug–disease relationships but also suggesting candidate drugs for which evidence has not yet been found.

## 4 Discussion

As a promising drug discovery strategy, DR can effectively solve the time-consuming and laborious problem of new drug discovery. In this study, we proposed a pretraining and fine-tuning DR model based on drug expression perturbation and drug effectiveness. To evaluate the prediction performance of DrugRepPT, we conducted a comprehensive comparison experiment, and the results demonstrated that DrugRepPT outperforms existing DR methods in terms of prediction accuracy.

The excellent performance of our DrugRepPT is attributed to several aspects. First, the expression information of genes under drug intervention is important for new drug discovery. In DrugRepPT, we design a pretraining module based on GCL, which takes the gene expression perturbation under drug intervention as the initial feature, alleviating the problem of sparse drug–disease relationship data by constructing a large-scale heterogeneous graph. In the end, the pretraining module generate features that integrated large-scale interactions between diseases and drugs and gene expression data. Second, previous DR methods mainly consider the binary relationships between drugs and diseases as the supervised information for the training model, but ignore the more important strong or weak relationship of drug effectiveness, e.g. irsogladine is more effective than omeprazole in treating peptic ulcer (Kuramoto *et al.* 2013). These methods also fail to integrate the binary relationships between drugs and diseases with ECR. We construct a fine-tuning module of graph residual-like convolution network, which captures more precise interactions among drugs and diseases, then introduce Bayesian multiloss to balance effectively the two relationships (binary relationships between drugs and diseases and ECR), finally achieving the training and predicting of DR model. Comprehensive experimental results also show that these properties help DrugRepPT significantly outperform all baseline methods in the DR task.

There remains some work to be done in the future. First, consensus signatures, an average of multiple experiments to summarize the effects of perturbations on CMap, show a pervasive similarity bias, which is an important potential confounder of analysis of perturbational datasets (Yue and Dutta 2022). In addition, the biological mechanisms underlying drug treatment of diseases are highly complex (Mitchell *et al.* 2023), and it is not enough to use only differential gene expression to reflect the dynamic changes at the molecular level; in the future, we hope to consider more changes at the molecular level (e.g. changes in RNA expression) to improve the richness and accuracy of the features of drug expression (Celaj *et al.* 2023). Second, when constructing large-scale heterogeneous networks, in addition to the two types of nodes and their relationships among drug and disease considered in our study, to capture the complex mechanisms of drug treatment diseases, more types of nodes (such as targets, symptoms, pathways, etc.) will be considered in the future to obtain a more comprehensive representation, to achieve more reliable DR prediction. Third, we effectively balance the existence and effectiveness of drug treatment by Bayesian multiloss; however, the model is still designed for a single-objective task, i.e. to predict the drug associated with the disease. Since the process of drug treatment for diseases is closely related to molecular interactions such as genes of diseases, targets of drugs, and PPIs (Loscalzo 2023), in the future, we will try to construct multitarget learning models to realize the simultaneous prediction of biomedical relationships such as disease–drug, drug–target, disease–gene, and protein–protein. This multitask learning can help alleviate the data sparsity of different types of relationships, achieve complementarity between feature representation and goal design, and ultimately improve the model’s generalization ability (Vandenhende *et al.* 2020).

## Author contributions

Shuyue Fan (Data curation, Methodology, Formal analysis, Programming, software development, Writing—original draft), Kuo Yang (Conceptulization, Methodology, Writing—review & editing), Kezhi Lu (Writing—review & editing), Xin Dong (Data curation), Xianan Li (Data curation), Qiang Zhu (Writing—review & editing), Shao Li (Writing—review & editing), Jianyang Zeng (Writing—review & editing), Xuezhong Zhou (Conceptulization, Writing—review & editing)

## Supplementary Material

btae692_Supplementary_Data

## Data Availability

The data underlying this article are available at https://github.com/2020MEAI/DrugRepPT.
