# Supplementary material for: DrugRepPT: a deep pretraining and fine-tuning framework for drug repositioning based on drug’s expression perturbation and treatment effectiveness
Source: Bioinformatics. 2024 Nov 19;40(12):btae692. doi: 10.1093/bioinformatics/btae692 (PMC11630837; doi:10.1093/bioinformatics/btae692)
Supplement: btae692_Supplementary_Data [file btae692_supplementary_data.zip › Supplementary_Materials_1.pdf]

Supplementary Materials for
DrugRepPT: a deep pre-training and fine-tuning framework for drug repositioning based on drug's expression perturbation and treatment effectiveness

1 Supplemental methods

1.1 Dataset

1.1.1 Indication-oriented drug effectiveness comparative data

In our prior work, namely, DRONet [1], we established a dataset of ECR among drugs through a combination of literature mining and hierarchical extension techniques. Specifically, we extracted drug and disease-related literature from the PubMed database [2] and ECR among drugs from the SemMed database [3], resulting in a comprehensive dataset comprising 3738 drug-disease relationships, encompassing 632 drugs and 264 diseases, each annotated with information on ECR. The ECR among drugs encompasses various types: higher than, same as, and lower than.

In this study, to obtain a more accurate ECR among drugs, we first restricted the types of ECR and curated 561 ECR among drugs in disease treatment. Subsequently, leveraging the drugs cataloged in the CMap [4], we identified 234 additional drug relationships. Employing hierarchical extension techniques from DRONet further augmented our dataset, enabling the discovery of additional relationships. For example, if drug A is found to be more effective than drug B in treating disease C, and drug B is more effective than drug C in treating the same disease, then we infer that drug A is more effective than drug C. Through this iterative process, we expanded our dataset to encompass a total of 418 ECR among drugs, enhancing the granularity and accuracy of our analysis.

1.1.2 Large-scale Drug-Disease Heterogeneous Graph

We have established a comprehensive drug-disease heterogeneous graph (DDHG) encompassing three kinds of relationships: drug-disease, drug-drug, and disease-disease, comprising a total of 21,858 drugs, 2,886 diseases, and 617,417 relationships (Figure 1). The process of collecting and organizing these relationships unfolded as follows.

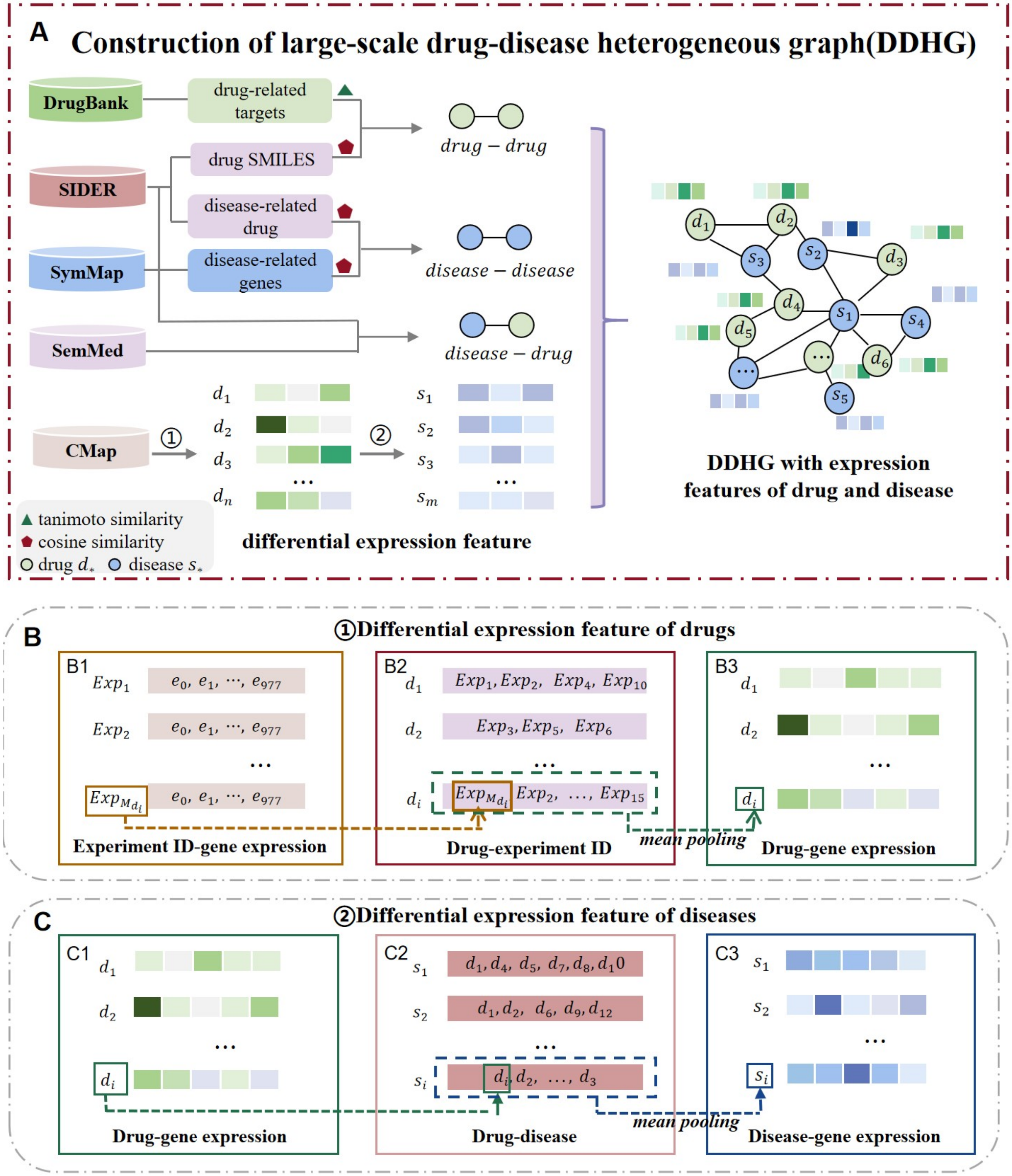

Figure 1: Construction of large-scale drug-disease heterogeneous graph (DDHG) (A) Construction of large-scale drug-disease heterogeneous graph with three relationship types and differential expression features. (B) Construction of the differential gene expression of the drugs. (C) Construction of the differential gene expression of the diseases.

Disease-drug relationships. We initially collected 30,835 disease-drug relationships from Side Effect Resource SIDER [5]. Subsequently, leveraging data from SemMed [3], we obtained 3,738 disease-drug relationships. Finally, the integration of these two sources yielded a comprehensive dataset comprising 18,297 disease-drug relationships, including 2,884 diseases and 2,046 drugs (Figure 1A).

Drug-drug relationships. Data originated from SIDER and DrugBank databases [6]. To ensure reliability, we employed drug similarity measures based on structural and molecular features, and then screened the drug-drug relationships with reliability greater than a given threshold. Specifically, we first extracted 1,414 drugs and their corresponding SMILES features from SIDER, and then calculated the Tanimoto similarity [7] of two-by-two drugs using the Python library (i.e., RDKit) and filtered 477,187 drug-drug relationships with greater than average similarity (=0.2091). Second, we collected drug-related targets from DrugBank and calculated target-based drug similarity based on cosine similarity, then reserved 3,247 drug-drug relationships with a threshold of 0.5. Integration of these two approaches yielded a total of 480,416 drug-drug relationships (Figure 1A).

Disease-Disease relationships. Data were sourced from SIDER and SymMap databases[8]. To obtain reliable disease-disease relationships, we calculated disease similarity based on drug and genetic information of the diseases respectively, and screened for disease-disease relationships whose reliability was greater than a given threshold. Specifically, we obtained the diseases and their associated drug from SIDER, calculated the disease similarity based on drug information using cosine similarity, and retained 112,393 disease relationships with similarity greater than the mean (=0.0039). Second, we collected diseases and their related genes from the SymMap, and also calculated disease similarity based on gene information using cosine similarity, retaining 6,557 disease-disease relationships with similarity greater than 0.5. Finally, a total of 118,704 disease-disease relationships were obtained by integrating the two types of relationships (Figure 1A).

1.1.3 Differential gene expression under drug intervention

Given that a drug used to treat diseases is intricately linked to the expression or suppression of relevant genes, understanding gene expression levels is paramount. To establish the features of drugs and diseases for DR modeling and subsequently enhance the precision of DR tasks, we leveraged gene expression perturbations observed under drug intervention from the Connectivity Map (CMap) database.

CMap, a perturbation transcriptomics database, employs various reagents to induce alterations in cell lines, subsequently analyzing transcriptomics data to generate a differential gene expression profile following reagent intervention. Perturbagen-cell line experiments within CMap encompass three primary categories: small compounds, gene knock-downs, and overexpressions. To optimize efficiency, CMap employs the L1000 assay platform to sequence 978 of these representative landmark genes. Additionally, 11,350 additional genes are inferred from these landmark genes using a vast collection of Affymetrix gene chips as training data. The CMap undergoes preprocessing by the Broad Institute, resulting in five distinct levels of data refinement. Notably, Level 3 data comprise 978 landmark genes and inferred expression values for the remaining genes.

Level 4 data encapsulate differential expression values relative to control profiles for each perturbation. Finally, Level 5 data represent merged and normalized profiles derived from replicated experiments.

In this study, we focused on Level 5 data, which contains 1,319,138 signatures, corresponding to 25,200 biological entities and 473,647 experimental records. Subsequently, we meticulously screened and extracted 30,632 records pertaining to 19,811 compounds from this dataset. These records include essential details such as experiment ID, and cell ID, as well as intervention specifics including compound ID, name, concentration, type, and duration, thus providing a comprehensive foundation for our research endeavors.

1.2 Graph contrastive neural network for pre-training

1.2.1 Gene expression features of drugs and diseases

We obtained differential gene expression generated under drug intervention from the CMap, which improved the reliability of the features of drug and disease by emphasizing the mechanism of action when drugs treat disease (Figure 1B and 1C). Specifically, for the drugs recorded in CMap, their differential gene expression can be obtained directly; for the drugs not recorded in CMap, we indirectly generated the differential gene expression of the drugs by establishing the relationships between these drugs and the recorded drugs through the above drug similarity; for the drug-related diseases, we generated the differential gene expression of the diseases based on the known disease-drug relationships.

Matching of drug and experimental ID. We screened the experimental data with the type of reagent that interfered with the cells as a compound, and obtained the experiment IDs of these drugs by matching with the drug's name in DDHG we constructed (Figure 1B1). Drug perturbation cell line experiments will be repeated several times, and the number of experimental records corresponding to each drug varies. M_d represents the number of experimental records corresponding to drug d, then the set of experimental records Exp_d corresponding to the drug d is represented as follows:

$$Exp_d = \{Exp_1, Exp_2, \ldots, Exp_{M_d}\} \tag{1}$$

Matching of experiment ID and differential gene expression. CMap tests for each of the drug-intervened cell line experiments to obtain the 978-dimensional differential gene expression caused by the drug reagents relative to normal cell lines (Figure 1B2). Differential gene expression F_{Exp} for each experiment ID Exp is shown as follows:

$$F_{Exp} = \{e_1, e_2, \ldots, e_{977}\} \tag{2}$$

Drug's features with differential gene expression. Since a drug corresponds to multiple experimental and each cell treated with the experiment will measure 978-dimensional differential gene expression values, the drug's differential gene expression is obtained by using average pooling of the differential gene expression under the multiple drug intervention experiments (Figure 1B3). Then the differential gene expression F_{d_r} of

the recorded drug d_r is expressed as follows:

$$F_{d_r} = \frac{\Sigma_{k=1}^{M_{d_r}} F_{Exp}^{k}}{M_{d_r}} \tag{3}$$

For the drugs not recorded in CMap, we obtained a set of associated recorded drugs corresponding to 2,047 unrecorded drugs based on the drug-drug relationships, and then for each unrecorded drug, we selected the top 10 recorded drugs with the highest similarity and performed average pooling on the gene expression features of the associated recorded drugs to obtain the final features of unrecorded drug. M_{d_n} represents the number of associated recorded drugs corresponding to unrecorded drugs d_n. Differential gene expression F_{d_n} corresponding to the unrecorded drugs d_n is shown as follows:

$$F_{d_n} = \frac{\Sigma_{k=1}^{M_{d_n}} F_{d_n}^{k}}{M_{d_n}} \tag{4}$$

Disease's features with differential gene expression. We obtained a set of associated drugs corresponding to 2,886 diseases based on the disease-drug relationship (Figure 1C2), and then performed average pooling on the gene expression features of the associated drugs for each disease to obtain the final disease's features (Figure 1C3). M_s represents the number of associated drugs corresponding to disease s. Differential gene expression F_s corresponding to the disease s is shown as follows:

$$F_s = \frac{\sum_{k=1}^{M_s} F_d^k}{M_s} \tag{5}$$

1.2.2 Graph contrastive neural network for pre-training

The GCL model consists of three key components: graph data augmentation (GDA), view encoders, and contrastive loss.

Graph data augmentation (GDA) GDA considers two strategies for dealing with graph corruption: structural hierarchy and attribute hierarchy, to achieve data augmentation. Structural hierarchy refers to removing edges for topology, that is, removing edges in the adjacency matrix A with the probability p_e to obtain the adjacency matrix after view augmentation. Attribute hierarchy refers to masking features for node attributes, that is, masking features with a probability p_f of zero for each node feature in the feature matrix X obtaining the features matrix X_* after view augmentation. Two new views, $\mathcal{G}_1$ and $\mathcal{G}_2$, are generated by breaking the original graph at the level of structure and attributes to build different node contexts for comparison with the original graph.

View encoders This component takes the classic GNN as a view encoder and formalizes each layer of the GNN into two processes, propagation and transformation, and uses three model augmentation techniques to improve the robustness of the encoder to obtain a node embedding representation of each view. The

propagation process of GNN is to calculate the graph filter F and the node feature Z to get the operators of propagation g; the transformation process is to calculate the node feature Z and parameter matrix W to get the operators of transformation h; finally, it is obtained through nonlinear transformation, as follows:

$$g(Z; F) = FZ, h(Z; W) = \sigma(ZW) \tag{6}$$

Model augmentation strategies include asymmetric strategy (using two contrast encoders with different numbers of propagation operators), random strategy (randomly varying the number of propagation operators at every epoch), and shuffling strategy (shuffling the permutation of propagation and transformation operators in the two view encoders). Formally, if the view encoder f has L propagation operators of g and N transformation operators of h, then two augmented view encoders be written as:

$$\begin{aligned} f_1 &= h_N \circ g_1^{[K_N]} ... h_1 \circ g_1^{[K_1]}, \\ f_2 &= h_N \circ g_2^{[K'_N]} ... h_1 \circ g_2^{[K'_1]} \end{aligned} \tag{7}$$

where $L = \sum_{i=1}^{N} K_i \neq \sum_{i=1}^{N} K_i^{'} = L^{'}$, and $\forall i K_i \neq K_i^{'}$.

Finally, node embedding in the two views generated by encoders f_1 and f_2 are obtained respectively, as follows:

$$Z_1 = f_1(X_1, A_1), Z_2 = f_2(X_2, A_2) \tag{8}$$

Contrastive loss Contrastive loss (CL) is defined as that for the node v_i, the embedding Z_1^i it generated in one view is treated as anchors, the embedding Z_2^i generated by v_i in another view is a positive sample, and nodes other than v_i in two views are regarded as negative samples [9]. We defined the pairwise objective for each positive pair (Z_1^i, Z_2^i) as:

$$\ell(Z_1^i, Z_2^i) = log \frac{e^{\varphi_1}}{e^{\varphi_1} + \sum_{k=1}^{N} \mathbb{I}_{[k \neq i]} e^{\varphi_2} + \sum_{k=1}^{N} \mathbb{I}_{[k \neq i]} e^{\varphi_3}} \tag{9}$$

where $\mathbb{I}_{[k \neq i]}$ is an indication function that equals to 1 if $k \neq i$. $\varphi_1 = \theta(Z_1^i, Z_2^i)/\tau, \varphi_2 = \theta(Z_1^i, Z_2^k)/\tau, \varphi_3 = \theta(Z_1^i, Z_1^k)/\tau$, τ is a temperature parameter, $\theta(Z_1, Z_2) = s(g(Z_1), g(Z_2))$, s is the cosine similarity and g is a non-linear projection. The first terms in the numerator and denominator represent positive pairs, and the second and third terms in the denominator represent negative pairs from inter-view and intra-view respectively. Since the two views are symmetric, the loss of the other view is similarly defined for $\ell(Z_2^i, Z_1^i)$. Finally, the overall objective function of the model is defined as the average of all positive pairs, as follows:

$$\mathcal{J} = \frac{1}{2N} \sum_{i=1}^{N} \left[\ell(Z_1^i, Z_2^i) + \ell(Z_2^i, Z_1^i) \right] \tag{10}$$

where N is the number of nodes. Based on this objective function, GCL can be trained and optimized. Subsequently, the model outputs embedding representation vectors for all drugs and diseases, which are then provided to the downstream DR fine-tuning model.

1.3 Bayesian Multi-loss Fine-tuning for drug repositioning

1.3.1 Residual-like graph convolution network for fine-tuning

Specifically, the input layer of the neural network is the embedding matrix of diseases and drugs obtained by the pre-training model, i.e., $E \in \mathbb{R}^{(M+N)\times D}$, with the first M rows of the matrix, i.e., $E_{[1:M]}$ is the disease embedding sub-matrix, and the last N rows of the matrix, i.e., $E_{[M:M+N]}$ is the drug embedding sub-matrix. The input layer of the neural network is $E^0 = E$. In the feature aggregation of (k+1)-th layer, the embedding of disease s_u and drug d_i are obtained by linear aggregation of the neighbor nodes of s_u and d_i in k-th layer (including disease and drug) and the embedding of themselves, respectively. The updated embedding is as follows:

$$\begin{aligned}\overline{[E^{k+1}]_{s_u}} &= \overline{e_{s_u}^{k+1}} = \frac{1}{diag(s_u)} e_{s_u}^k + \sum_{d_j \in R_{s_u}} \frac{1}{diag(d_j) \times diag(s_u)} e_{d_j}^k \\ \overline{[E^{k+1}]_{d_i}} &= \overline{e_{d_i}^{k+1}} = \frac{1}{diag(d_i)} e_{d_i}^k + \sum_{s_u \in R_{d_i}} \frac{1}{diag(d_i) \times diag(s_u)} e_{s_u}^k\end{aligned} \tag{11}$$

which diag$(\cdot)$ is the diagonal degree of disease s_u (drug d_i) in the disease-drug bipartite graph G. R_* is neighbors of node $(*)$ in graph G.

The final embedding representation of diseases and drugs at the (k+1)-th layer, which is obtained by applying a nonlinear transformation to the aggregated features, is as follows:

$$\begin{aligned}[E^{k+1}]_{s_u} &= e_{s_u}^{k+1} = RELU[\overline{e_{s_u}^{k+1}} W^k] \\ [E^{k+1}]_{d_i} &= e_{d_i}^{k+1} = RELU[\overline{e_{d_i}^{k+1}} W^k]\end{aligned} \tag{12}$$

where $RELU$ is a non-linear activation function RELU.

In addition, to alleviate the over-smoothing effect with deeper layers, we employ residual-like learning, and then through the non-linear activation function RELU, we ultimately obtain the score value for drug treatment of diseases, representing the potential likelihood of ECR among drugs in treating diseases,

$$\hat{r}_{ui}^{k+1} = RELU(\hat{r}_{ui}^{k} + < e_{s_u}^{k+1}, e_{d_i}^{k+1} >) \tag{13}$$

where $<,>$ denotes vector inner product operation.

1.4 Prediction of molecules with drug potential

In the field of drug development, related researchers have discovered and synthesized a large number of molecules with potential drug activity through chemical and biosynthetic methods [10; 11; 12]. These molecules may be able to treat, prevent, or diagnose disease and are an important building block for modern drug discovery. Our framework can also identify molecules with drug potential, that is, it can predict the score between a disease D and a candidate drug S if S is not in DDHG. Because molecules have chemical structures, we can employ drug similarity measures based on chemical structural features to calculate the similarity between unknown molecule S and known drugs (drugs in DDHG). Then we can screen the reliable

drug-drug relationships with a proper threshold, and then link the molecule S to the known drugs in DDHG. Specifically, we extract the SMILES features of a drug candidate S not in DDHG and drugs in DDHG from SIDER and then use the Python library (i.e. RDKit) to calculate the Tanimoto similarity between S and drugs in DDHG, preserving the relationships of greater than average similarity. When the drug candidate S is linked to the known drugs in DDHG, it means that the drug candidate is added to DDHG, and then our method could measure the possibility that the drug candidate treats different diseases.

2 Supplemental experimental setting

2.1 Training and test data set

In the experiment, we divided 2,656 drug-disease relationships into the training and test set according to the ratio of 8:2. When generating negative samples, we first constructed all the relationships between each drug and each disease, then removed the existing relationships between drug and diseases, finally randomly selected the samples with specified quantity from the rest relationships as negative samples.

2.2 Baseline methods for comparison

In the comparison experiment, we adopted two types of baseline methods: 1) existing drug repositioning methods; and 2) prediction methods based on embedding feature similarity. We selected several existing drug repositioning methods (i.e., LAGCN [13], NIMCGCN [14], HNet-DNN [15] and DRONet [1]) with public source codes. LAGCN integrates known drug-disease associations, drug-drug similarities, and disease-disease similarities into a heterogeneous network and applies graph convolution operation to the network to the embedding of drugs and diseases. The NIMCGCN model combines miRNA-miRNA and disease-disease similarity networks, employing graph convolution networks (GCN) to capture their feature representations, and uses neural inductive matrix completion to infer unknown drug-disease associations. HNET-DNN extracts the underlying features of drug and disease from drug-disease heterogeneous networks and constructs a deep neural network to predict drug indications. DRONet makes full use of ECR among drugs by combining network embedding and ranking learning to learn the representation vector of drug and disease from heterogeneous networks and designed a ranking learning model to predict drug-disease relationships.

In the prediction methods based on the embedding feature similarity, we first conducted NE algorithms to obtain the embedding features from drugs and diseases and utilized cosine similarity to calculate the similarity of the embedding features between drugs and diseases. The greater the similarity means the stronger the relevance of drug-disease relationships. In the experiments, we used three types of NE methods: 1) matrix decomposition related methods, including SVD, HOPE [16], GF [17], GraRep [18]; 2) random walk related methods, including DeepWalk [19] and node2vec [20]; 3) neural network related methods, including LINE [21], SDNE [22] and GAE [23].

The input features of the above baseline methods include two types (Table 1). One is the drug-disease heterogeneous network. For example, the prediction methods based on embedding feature similarity (i.e., HOPE, SVD, GraRep, Deepwalk, GF, Node2vec, SDNE and LINE), LAGCN, NIMCGCN and DRONet all use drug-disease heterogeneous networks as input features. The others are chemical structures combined with drug-disease heterogeneous networks. For example, HNet-DNN obtains SMILES string from the PubChem database and the medical subject headings (MeSH) terms as the initial features of drugs and diseases.

To evaluate the effect of input features on the performance of the baseline methods, we analyzed their ablation experiments (AE). The AE results of HNet-DNN showed that the features extracted from the heterogeneous network significantly improved performance compared to ontology features. The AE results of DRONet (i.e., $DRONet_{RN}$, $DRONet_{LM}$ and $DRONet_{LR}$) showed that the features extracted from the heterogeneous network contribute more to performance than learning to rank. However, LAGCN and NIMCGCN did not conduct ablation experiments to evaluate the impact of features on performance. Furthermore, the prediction methods based on embedding feature similarity (i.e., SVD, HOPE, GF, GraRep, DeepWalk, node2vec, LINE and SDNE) used the network embedding algorithm to obtain the features of drugs and diseases from the drug-disease heterogeneous network, and then calculated the similarity between diseases and drugs using the Euclidean distance to predict diseases-drugs relationships. Therefore, the model cannot be trained when removes the drug-disease heterogeneous network.

Table 1: Comparison of input features for baseline methods

DR methods	Drug-disease heterogeneous network	SMILES	MeSH
HOPE	√		
SVD	√		
GraRep	√		
Deepwalk	√		
GF	√		
Node2vec	√		
SDNE	√		
LINE	√		
LAGCN	√		
HNet-DNN	√	√	√
NIMCGCN	√		
$DRONet_{RN}$	√		
$DRONet_{LM}$	√		
$DRONet_{LR}$	√		

2.3 Evaluation metrics

In the experimental evaluation of drug repositioning, we selected Mean Reciprocal Rank (MRR) and top K hit ratio (Hit@K) as evaluation metrics and evaluated the performance improvement of DrugRepPT by using the relative improvement rate.

MRR can evaluate the position of the true associated drug in the list of candidate drugs for the disease.

$$MRR = \frac{\sum_{i=1}^{M} \sum_{s_i=1}^{N_{s_i}} \frac{1}{rank_{s_i}}}{\sum_{i=1}^{M} N_{s_i}} \tag{14}$$

where $rank_{s_i}$ is the position of the true associated drug in the predicted candidate list for disease s_i, N_{s_i} represents the number of the truly associated drugs for disease s_i, and M is the number of diseases.

Hit@K can evaluate the proportion of top-K drugs in the candidate drug list among the real drugs, the formula is as follows.

$$Hit@K = \frac{\sum_{i=1}^{M} \sum_{j=1}^{N_{s_i}} top(K)}{\sum_{i=1}^{M} N_{s_i}} \tag{15}$$

where, $top(K)$ indicates whether the true associated drug for disease s_i is in the top-K of the candidate list; if so, it is 1; otherwise, it is 0; M is the number of diseases; N_{s_i} is the number of the true associated drug for disease s_i.

The relative improvement rate is defined as the percentage increase in an evaluation metric achieved by DrugRepPT compared to baseline methods, the formula is as follows.

$$Improvement = \frac{EM_{DrugRepPT} - EM_{baseline}}{EM_{baseline}} \times 100\% \tag{16}$$

Where EM_* indicates the evaluation metrics obtained by method $(*)$.

2.4 Parameter settings

DrugRepPT is implemented with PyTorch, and the entire model is trained on GeForce RTX 3090Ti GPU with 500GB of memory. In our experiments, we carried out an extensive grid search, over the following ranges of hyper-parameter values: position-aware weights β in $\{10^{-3}, 10^{-2}, 10^{-1}, 10^{0}, 10^{1}, 10^{2}\}$, learning rate in $\{0.001, 0.005, 0.01, 0.05, 0.1\}$, batch size in $\{2^8, 2^{10}, 2^{12}, 2^{14}, 2^{16}\}$ and GCN layer in $\{1, 2, 3, 4, 5\}$.

3 Supplemental results

3.1 Ablation experiments of DrugRepPT

3.1.1 Contribution of different heterogeneous networks of DrugRepPT

In the process of learning the features of drugs and diseases through heterogeneous networks, the network structures with different relationships have effects on the node features to be learned. Therefore, we constructed four drug-disease heterogeneous networks, including different relationships (Table 2).

On the whole, the results show that there is a slight performance difference between DrugRepPT with different networks. Except for the terms of Hit@10, $DDHG_E$ containing drug-disease relationships obtained

Table 2: Performance comparison of drug repositioning methods

DR methods	Hit@1	Hit@3	Hit@10	MRR
[a] $DDHG_A$	0.2466±0.0025	0.4809±0.0035	0.7475±0.0029	0.4058±0.0019
[b] $DDHG_B$	0.2461±0.0021	0.4877±0.0029	0.7554±0.0018	0.407±0.0014
[c] $DDHG_C$	0.2387±0.0019	0.4952±0.0046	0.7633±0.0016	0.4074±0.0019
[d] $DDHG_D$	0.0194±0.004	0.0387±0.0013	0.0952±0.0022	0.0644±0.0023
[e] $DDHG_E$	0.0196±0.0027	0.0453±0.006	0.1341±0.0085	0.0711±0.0035

[a] a comprehensive drug-disease heterogeneous graph (DDHG) encompassing three kinds of relationships: drug-disease, drug-drug and disease-disease.

[b] $DDHG_A$ removes drug-drug relationships with a similarity bigger than 0.5.

[c] $DDHG_A$ removes disease-disease relationships with a similarity bigger than 0.5.

[d] $DDHG_A$ removes drug-drug and diseases-diseases relationships with a similarity bigger than 0.5.

[e] $DDHG_A$ removes drug-drug and diseases-diseases relationships.

the best performance. The drug's high similarity relationships in the network partially improve the performance of DrugRepPT, namely $DDHG_A$ performs on Hit@1 and Hit@10 is higher than $DDHG_B$. From the term of Hit@10, the disease's high similarity relationships have a negative influence and reduce the performance of the model, namely, $DDHG_A$ performs on Hit@10 lower than $DDHG_C$. However, as measured by Hit@10, the high similarity between drugs and high similarity between diseases work together that improve the performance of DrugRepPT. Therefore, the performance differences of network structures with different relationships are more like fluctuations at the experimental level than the effects of their relationships. In summary, drug-drug relationships and disease-disease relationships with the high similarity contributed little to improve model performance.

3.2 Hyper-parameter sensibility analysis

3.2.1 Impact of Different β

In the objective function we designed, an important hyper-parameter is β, which is actually a threshold. We compared the effect of β on predictive performance (Figure 2A1–A4). The experimental results show that when β decreases, DrugRepPT performance increases, and when β increases, DrugRepPT performance decreases, which indicates that the binary relationships between drugs and diseases plays a greater role than ECR among drugs in the DR task. However, the results of the ablation experiment also indicate that ECR among drugs also plays a certain role. There may be two reasons for this phenomenon: first, the existing drug effectiveness comparative data are still few, resulting in the performance improvement of the model is not obvious; second, in the performance evaluation of the DR task, the association between drug and disease is used as the test set, so the evaluation index does not evaluate the ability of the model to predict ECR among drugs, but focuses more on the ability to predict new potential drugs for diseases.

3.2.2 Impact of Different learning rate

We compared the effect of learning rate on predictive performance (Figure 2B1–B4). When the learning rate is 0.1, DrugRepPT obtains the highest predicted performance. As the learning rate decreases, the performance decreases first and then increases.

3.2.3 Impact of Different batch size

We compared the effect of batch size on predicted performance (Figure 2C1–C4). When the batch size is 2^{16}, DrugRepPT achieves the highest predicted performance, which is equivalent to the entire training set for each batch. With the decrease in batch size, the performance shows a downward trend. When the whole training set is used directly, the model has the best effect without the over-fitting phenomenon, which may be because the input of the residual GCN-based fine-tuning model is a small graph (625 nodes and 2656 edges). At the same time, the data on the drug-disease relationship and ECR among drugs are also small.

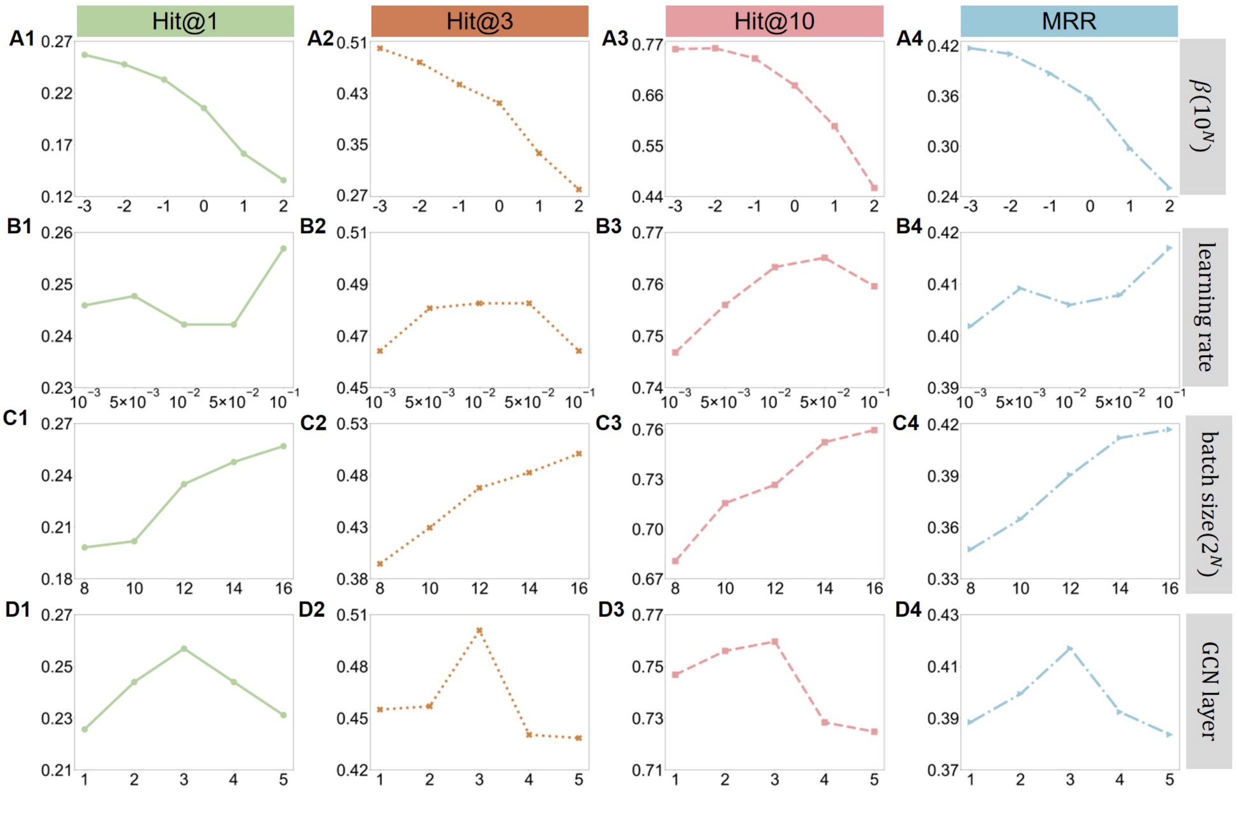

Figure 2: Parameter sensitivity analysis results of DrugRepPT. (A1–A4) DrugRepPT's performance with different position-aware weights β. (B1–B4) DrugRepPT's performance with different learning rates. (C1–C4) DrugRepPT's performance with different batch sizes. (D1–D4) DrugRepPT's performance with different GCN layers.

3.2.4 Impact of Different GCN layer

We compared the effect of the number of GCN layers on the predictive performance of DrugRepPT (Figure 2D1–D4). The results shows that with the increase in the number of GCN layers, the performance first increases and then decreases. The reason for the improvement of the model performance may be that the complexity of the model increases with the increase of the number of GCN layers, and the topology of the graph can be learned better accordingly. The reason for the deterioration of the model performance may be that too many layers of the neural network will lead to excessive information transmission between nodes, resulting in more similar features of the nodes, and the problem of over-smoothing of the neural network.

3.3 Case analysis of fatty liver

3.3.1 Validation of biomedical literature

We retrieved the newly published biomedical literature from PubMed and evaluated the top 10 drugs predicted by DrugRepPT for fatty liver (Table 3 of Manuscript).

The results of retrieving biomedical literature shows four drugs that are mentioned in the newly published literature about the treatment of fatty liver. Carroll et al. proposed tacrolimus (rank=2 in DrugRepPT) is a safe and well-tolerated treatment for Autoimmune hepatitis [24]. Malladi et al. found that the use of aspirin, ticlopidine (rank=4 in DrugRepPT), and cilostazole could help control the progression of non-alcoholic fatty liver disease [25]. Abdallah et al. investigated the efficacy and safety of montelukast (rank=5 in DrugRepPT) in the treatment of patients with non-alcoholic fatty liver disease [26]. Porteiro et al. found that low-dose of doxorubicin (rank=7 in DrugRepPT) had beneficial effects on nonalcoholic fatty liver disease and nonalcoholic steatohepatitis [27].

In addition, we retrieved the newly published biomedical literature from PubMed and evaluated the top 10 drugs predicted by $DRONet_{LR}$ for gastritis and fatty liver (Table 3).

First, the results shows that five drugs are mentioned in newly published literature in relation to the treatment of gastritis. Specifically, Kharbanda et al. analyzed medical records and found that patients with granulomatous gastritis were able to significantly improve renal function and gastrointestinal symptoms with oral prednisone (rank=1 in $DRONet_{LR}$) [28]. Quentin et al. found that an empirical antibiotic therapy with piperacillin/tazobactam and amikacin (rank=3 in $DRONet_{LR}$) alleviated necrotizing esophagitis and gastritis caused by hypervirulent PVL positive ST 121 CA-MRSA through clinical cases [29]. Dahal et al. found that the most common infections treated with third-generation cephalosporins cefixime (rank=5 in $DRONet_{LR}$) were acute gastritis [30]. Matis et al. found that the administration of vancomycin and piperacillin-tazobactam in conjunction with clindamycin (rank=7 in $DRONet_{LR}$) improved the clinical presentation of emphysematous gastritis [31]. Moezi et al. found that pioglitazone (rank=8 in $DRONet_{LR}$) exhibits gastroprotective actions [32].

The results of retrieving biomedical literature shows that four drugs that are mentioned in newly published literature in relation to the treatment of fatty liver. Abdallah et al. investigated the efficacy and safety

Table 3: **Top 10 candidate drugs predicted by $DRONet_{LR}$ for gastritis and fatty liver**

Rank	Gastritis	Fatty liver
1	[b]prednisone	olanzapine
2	lamotrigine	clarithromycin
3	[b]amikacin	[b]montelukast
4	[a]omeprazole	ceftazidime
5	[b]cefixime	[b]tacrolimus
6	rofecoxib	[a]rosiglitazone
7	[b]clindamycin	sulfasalazine
8	[b]pioglitazone	tigecycline
9	tramadol	[b]doxorubicin
10	guanfacine	ciclopirox

[a] These candidate drugs predicted by DR models are in the test set.

[b] Several newly published literature reported that these candidate drugs are possibly related to the treatment of gastritis and fatty liver.

of montelukast (rank=3 in $DRONet_{LR}$) in the treatment of patients with non-alcoholic fatty liver disease [26]. Carroll et al. proposed tacrolimus (rank=5 in $DRONet_{LR}$) as a safe and well-tolerated treatment for Autoimmune hepatitisAIH [24]. Porteiro et al. found that low-dose of doxorubicin (rank=9 in $DRONet_{LR}$) had beneficial effects on nonalcoholic fatty liver disease and nonalcoholic steatohepatitis [27].

3.3.2 Validation of network medicine

For fatty liver, we obtained 101 genes of disease in MalaCards and 75 targets for predicted drugs. The dense links (91 real links vs 16.58 expected links, P=1.91E-37, binomial test) that hold in the PPI network indicated that those two kinds of genes would tend to have closer interactions than expectation (Figure 5D of Manuscript).

3.3.3 Validation of docking-based virtual screening

There is strong binding energy with the protein PPARA related to fatty liver for the five predicted drugs, including pyrazinamide (ranked 3, BE=-4.64), clarithromycin (ranked 6, BE=-15.64), olanzapine (ranked 8, BE=-8.89), urapidil (ranked 9, BE=-7.47) and sulfasalazine (ranked 10, BE=-6.79). Taking the predicted drug pyrazinamide as an example, it docks onto the three amino acid residues of the 6LXA, namely LYS-399, PRO-295, and ARG-209 (Figure 5F of Manuscript).

References

1 Yang K, Yang Y, Fan S, Xia J, Zheng Q, Dong X, et al. DRONet: effectiveness-driven drug repositioning framework using network embedding and ranking learning. Briefings in bioinformatics. 2023;24(1):bbac518.

2 Lu Z. PubMed and beyond: a survey of web tools for searching biomedical literature. Database. 2011;2011:baq036.

3 Kilicoglu H, Shin D, Fiszman M, Rosemblat G, Rindflesch TC. SemMedDB: a PubMed-scale repository of biomedical semantic predications. Bioinformatics. 2012;28(23):3158-60.

4 Subramanian A, Narayan R, Corsello SM, Peck DD, Natoli TE, Lu X, et al. A next generation connectivity map: L1000 platform and the first 1,000,000 profiles. Cell. 2017;171(6):1437-52.

5 Kuhn M, Letunic I, Jensen LJ, Bork P. The SIDER database of drugs and side effects. Nucleic acids research. 2016;44(D1):D1075-9.

6 Wishart DS, Feunang YD, Guo AC, Lo EJ, Marcu A, Grant JR, et al. DrugBank 5.0: a major update to the DrugBank database for 2018. Nucleic acids research. 2018;46(D1):D1074-82.

7 Butina D. Unsupervised data base clustering based on daylight's fingerprint and tanimoto similarity: a fast and automated way to cluster small and large data sets. Journal of Chemical Information and Computer Sciences. 1999;39(4):747-50.

8 Wu Y, Zhang F, Yang K, Fang S, Bu D, Li H, et al. SymMap: an integrative database of traditional Chinese medicine enhanced by symptom mapping. Nucleic acids research. 2019;47(D1):D1110-7.

9 Zhu Y, Xu Y, Yu F, Liu Q, Wu S, Wang L. Deep graph contrastive representation learning. arXiv preprint arXiv:200604131. 2020.

10 Harrison SA, Allen AM, Dubourg J, Noureddin M, Alkhouri N. Challenges and opportunities in NASH drug development. Nature medicine. 2023;29(3):562-73.

11 Loewa A, Feng JJ, Hedtrich S. Human disease models in drug development. Nature reviews bioengineering. 2023;1(8):545-59.

12 Trajanoska K, Bhérer C, Taliun D, Zhou S, Richards JB, Mooser V. From target discovery to clinical drug development with human genetics. Nature. 2023;620(7975):737-45.

13 Yu Z, Huang F, Zhao X, Xiao W, Zhang W. Predicting drug–disease associations through layer attention graph convolutional network. Briefings in bioinformatics. 2021;22(4):bbaa243.

14 Li J, Zhang S, Liu T, Ning C, Zhang Z, Zhou W. Neural inductive matrix completion with graph convolutional networks for miRNA-disease association prediction. Bioinformatics. 2020;36(8):2538-46.

15 Liu H, Zhang W, Song Y, Deng L, Zhou S. HNet-DNN: inferring new drug–disease associations with deep neural network based on heterogeneous network features. Journal of Chemical Information and Modeling. 2020;60(4):2367-76.

16 Ou M, Cui P, Pei J, Zhang Z, Zhu W. Asymmetric transitivity preserving graph embedding. In: Proceedings of the 22nd ACM SIGKDD international conference on Knowledge discovery and data mining; 2016. p. 1105-14.

17 Ahmed A, Shervashidze N, Narayanamurthy S, Josifovski V, Smola AJ. Distributed large-scale natural graph factorization. In: Proceedings of the 22nd international conference on World Wide Web; 2013. p. 37-48.

18 Cao S, Lu W, Xu Q. Grarep: learning graph representations with global structural information. In: Proceedings of the 24th ACM international on conference on information and knowledge management; 2015. p. 891-900.

19 Perozzi B, Al-Rfou R, Skiena S. Deepwalk: online learning of social representations. In: Proceedings of the 20th ACM SIGKDD international conference on Knowledge discovery and data mining; 2014. p. 701-10.

20 Grover A, Leskovec J. Node2vec: scalable feature learning for networks. In: Proceedings of the 22nd ACM SIGKDD international conference on Knowledge discovery and data mining; 2016. p. 855-64.

21 Tang J, Qu M, Wang M, Zhang M, Yan J, Mei Q. Line: large-scale information network embedding. In: Proceedings of the 24th international conference on world wide web; 2015. p. 1067-77.

22 Wang D, Cui P, Zhu W. Structural deep network embedding. In: Proceedings of the 22nd ACM SIGKDD international conference on Knowledge discovery and data mining; 2016. p. 1225-34.

23 Kipf TN, Welling M. Variational graph auto-encoders. Neural Information Processing Systems Workshop on Bayesian Deep Learning. 2016;1050:21.

24 Carroll G, Wasson G, McDougall N, McCorry R, Cadden I, Cash J. PTH-084 The efficacy of tacrolimus as an alternative agent in the treatment of autoimmune hepatitis. Gut. 2018;67(Suppl 1):A120-1.

25 Malladi N, Alam MJ, Maulik SK, Banerjee SK. The role of platelets in non-alcoholic fatty liver disease: from pathophysiology to therapeutics. Prostaglandins Other Lipid Mediators. 2023;169:106766.

26 Abdallah MS, Eldeen AH, Tantawy SS, Mostafa TM. The leukotriene receptor antagonist montelukast in the treatment of non-alcoholic steatohepatitis: a proof-of-concept, randomized, double-blind, placebo-controlled trial. European Journal of Pharmacology. 2021;906:174295.

27 Porteiro B, Fondevila MF, Buque X, Gonzalez-Rellan MJ, Fernandez U, Mora A, et al. Pharmacological stimulation of p53 with low-dose doxorubicin ameliorates diet-induced nonalcoholic steatosis and steatohepatitis. Molecular metabolism. 2018;8:132-43.

28 Kharbanda S, Leung J, Prakash A, Madhotra R. P154 A complex case of granulomatous gastritis. Gut. 2023;72(Suppl 2):A136-6.

29 Richier Q, Pilmis B, Chhor V, Tran M, Bruel C, Philippart F. Necrotizing esophagitis and gastritis due to hypervirulent PVL positive ST 121 CA-MRSA. Journal of Travel Medicine. 2022;29(4):taac007.

30 Dahal S, Bhandari S, Ghimire B, Mahesh N, Krishnamurthy M, AK AK. Drug utilization evaluation of third generation cephalosporins in a tertiary care hospital. Therapy. 2017;11(12):13.

31 Matis M, Wiant T, Singh A, Gunter N. Emphysematous gastritis: the success of early diagnosis and conservative management. In: C53. CRITICAL CARE GASTROENTEROLOGY: CASES. American Thoracic Society; 2023. p. A5319-9.

32 Moezi L, Heidari R, Amirghofran Z, Nekooeian AA, Monabati A, Dehpour AR. Enhanced anti-ulcer effect of pioglitazone on gastric ulcers in cirrhotic rats: the role of nitric oxide and IL-1β. Pharmacological Reports. 2013;65(1):134-43.
