# Supplementary material for: DrugRepPT: a deep pretraining and fine-tuning framework for drug repositioning based on drug’s expression perturbation and treatment effectiveness
Source: Bioinformatics. 2024 Nov 19;40(12):btae692. doi: 10.1093/bioinformatics/btae692 (PMC11630837; doi:10.1093/bioinformatics/btae692)
Supplement: btae692_Supplementary_Data [file btae692_supplementary_data.zip › Table S3.docx]

Table S3. Top 10 candidate drugs predicted by $\mathrm{DRONet}_{\mathrm{LR}}$ for gastritis and fatty liver

| **Rank** | **Gastritis** | **Fatty liver** |
| --- | --- | --- |
| 1 | **^b^prednisone** | olanzapine |
| 2 | lamotrigine | clarithromycin |
| 3 | **^b^amikacin** | **^b^montelukast** |
| 4 | **^a^omeprazole** | ceftazidime |
| 5 | **^b^cefixime** | **^b^tacrolimus** |
| 6 | rofecoxib | **^a^rosiglitazone** |
| 7 | **^b^clindamycin** | sulfasalazine |
| 8 | **^b^pioglitazone** | tigecycline |
| 9 | tramadol | **^b^doxorubicin** |
| 10 | guanfacine | ciclopirox |

**^a^** These candidate drugs predicted by DR models are in the test set.

**^b^** Several newly published literature reported that these candidate drugs are possibly related to the treatment of gastritis and fatty liver.
