# Supplementary material for: DrugRepPT: a deep pretraining and fine-tuning framework for drug repositioning based on drug’s expression perturbation and treatment effectiveness
Source: Bioinformatics. 2024 Nov 19;40(12):btae692. doi: 10.1093/bioinformatics/btae692 (PMC11630837; doi:10.1093/bioinformatics/btae692)
Supplement: btae692_Supplementary_Data [file btae692_supplementary_data.zip › Table S2.docx]

Table S2. Comparison of input features for baseline methods

| DR methods | **Drug-disease heterogeneous network** | **SMILES** | **MeSH** |
| --- | --- | --- | --- |
| **HOPE** | √ |  |  |
| **SVD** | √ |  |  |
| **GraRep** | √ |  |  |
| **Deepwalk** | √ |  |  |
| **GF** | √ |  |  |
| **Node2vec** | √ |  |  |
| **SDNE** | √ |  |  |
| **LINE** | √ |  |  |
| **LAGCN** | √ |  |  |
| **HNet-DNN** | √ | √ | √ |
| **NIMCGCN** | √ |  |  |
| $\boldsymbol{DRONet}_{\mathbf{RN}}$ | √ |  |  |
| $\boldsymbol{DRONet}_{\boldsymbol{LM}}$ | √ |  |  |
| $\boldsymbol{DRONet}_{\boldsymbol{LR}}$ | √ |  |  |
