# Supplementary material for: DrugRepPT: a deep pretraining and fine-tuning framework for drug repositioning based on drug’s expression perturbation and treatment effectiveness
Source: Bioinformatics. 2024 Nov 19;40(12):btae692. doi: 10.1093/bioinformatics/btae692 (PMC11630837; doi:10.1093/bioinformatics/btae692)
Supplement: btae692_Supplementary_Data [file btae692_supplementary_data.zip › Table S1.docx]

Table S1. The results of ablation experiments of different heterogeneous networks of DrugRepPT

| DR methods | hit@1 | hit@3 | hit@10 | MRR |
| --- | --- | --- | --- | --- |
| ^a^${DDHG}_{A}$ | 0.2466±0.0025 | 0.4809±0.0035 | 0.7475±0.0029 | 0.4058±0.0019 |
| ^b^${DDHG}_{B}$ | 0.2461±0.0021 | 0.4877±0.0029 | 0.7554±0.0018 | 0.407±0.0014 |
| ^c^${DDHG}_{C}$ | 0.2387±0.0019 | 0.4952±0.0046 | 0.7633±0.0016 | 0.4074±0.0019 |
| ^d^${DDHG}_{D}$ | 0.2439±0.0041 | 0.4734±0.004 | 0.7567±0.0025 | 0.4048±0.0016 |
| ^e^${DDHG}_{E}$ | 0.2589±0.0019 | 0.4971±0.0039 | 0.7582±0.0018 | 0.4181±0.001 |

^a^ a comprehensive drug-disease heterogeneous graph (DDHG) encompassing three kinds of relationships: drug-disease, drug-drug and disease-disease.

^b^ $\mathrm{DDHG}_{A}$ removes drug-drug relationships with a similarity bigger than 0.5.

^c^ $\mathrm{DDHG}_{A}$ removes disease-disease relationships with a similarity bigger than 0.5.

^d^ $\mathrm{DDHG}_{A}$ removes drug-drug and diseases-diseases relationships with a similarity bigger than 0.5.

^e^ $\mathrm{DDHG}_{A}$ removes drug-drug and diseases-diseases relationships.
