# Supplementary figures and images for: DrugRepPT: a deep pretraining and fine-tuning framework for drug repositioning based on drug’s expression perturbation and treatment effectiveness
Source: Bioinformatics. 2024 Nov 19;40(12):btae692. doi: 10.1093/bioinformatics/btae692 (PMC11630837; doi:10.1093/bioinformatics/btae692)

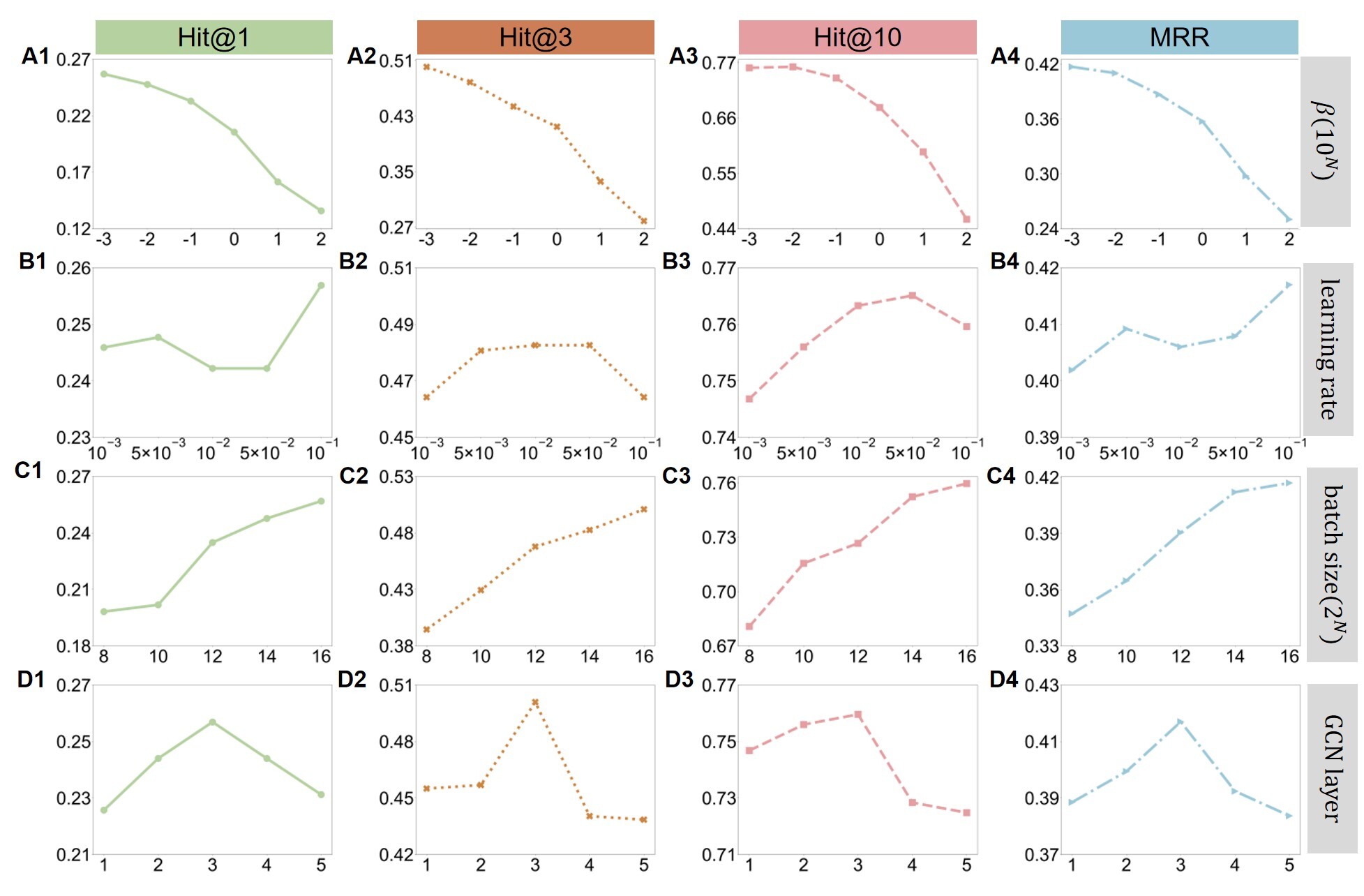

Supplement: btae692_Supplementary_Data [file btae692_supplementary_data.zip › figS2.jpg]

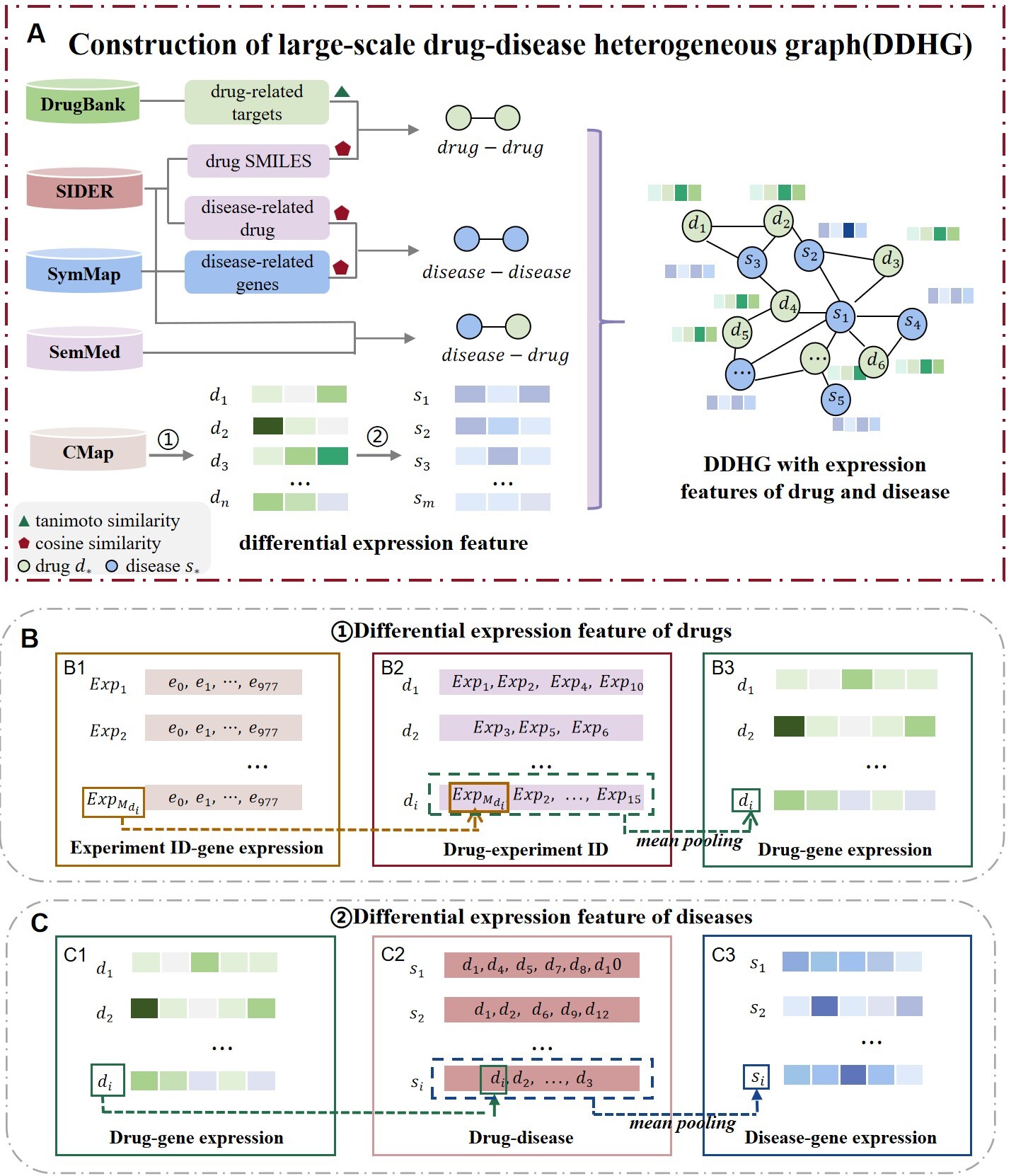

Supplement: btae692_Supplementary_Data [file btae692_supplementary_data.zip › figS1.jpg]
